# Sn‐Driven Grain Boundary Elimination for Single‐Crystalline Ni‐Rich Cathodes with Superior High‐Voltage Stability in Lithium‐Ion Batteries

**DOI:** 10.1002/advs.77769

**Published:** 2026-09-16

**Authors:** Guomeng Xie, Delai Qian, Ruiwu Li, Terence Xiaoteng Liu, Shun Lu

**Affiliations:** ^1^ School of Materials Science and Engineering Harbin Institute of Technology Harbin People's Republic of China; ^2^ School of Engineering, Physics and Mathematics, Faculty of Science and 11Environment Northumbria University Newcastle Upon Tyne UK; ^3^ Chongqing Institute of Green and Intelligent Technology Chinese Academy of Sciences Chongqing People's Republic of China

**Keywords:** grain‐boundary‐elimination approach, lithium‐ion batteries, low‐temperature strategy, Single‐crystalline Ni‐rich cathodes, Sn‐doping

## Abstract

Single‐crystalline Ni‐rich cathodes are regarded as promising candidates for high‐energy‐density lithium‐ion batteries (LIBs), providing superior mechanical and structural stability compared to polycrystalline counterparts. In this work, we present a low‐temperature grain‐boundary‐elimination approach to synthesize single‐crystalline LiNi_0.9_Co_0.08_Al_0.02_O_2_ (SC‐NCA) through Sn‐assisted modification. Beyond enabling low‐temperature synthesis, the integrated Sn serves a dual‐protective function. Bulk Sn doping robustly pins lattice oxygen and mitigates harmful irreversible phase transitions, preserving structural integrity. Simultaneously, the residual formation of a highly conductive Li_2_SnO_3_ surface layer accelerates Li^+^ diffusion kinetics and robustly passivates the cathode‐electrolyte interphase against parasitic degradation. Consequently, this dual‐site modified SC‐Sn‐NCA90 cathode exhibits exceptional electrochemical performance, delivering a remarkable capacity retention of 86.7% after 500 cycles at 1 C (2.8–4.5 V), vastly eclipsing the 40.8% retention of the polycrystalline benchmark. This work presents a low‐temperature strategy for synthesizing SC‐NCA combined with Sn doping, facilitating the development of Ni‐rich layered cathodes for high‐energy‐density LIBs.

## Graphical abstract

A low‐temperature synthesis method of single‐crystalline NCA was successfully achieved by Sn doping, employing the same synthetic conditions as its polycrystalline counterparts. This synthesis was conducted at a temperature that was 150 °C lower than that required for synthesizing single‐crystalline NCA without Sn doping. Density functional theory calculations and experimental observations demonstrate that Sn doping within the bulk of the grain effectively stabilizes the lattice and alleviates detrimental irreversible phase transitions. Meanwhile, the residual Li‐ion conductive Li_2_SnO_3_ on the grain surface enhances the Li^+^ diffusion kinetics and mitigates the parasitic reactions at high cut‐off voltages. Consequently, SC‐Sn‐NCA90 demonstrates excellent electrochemical performance, achieving a high capacity retention of 86.7% after 500 cycles at 1 C between 2.8–4.5 V, compared to only 40.8% for PC‐NCA90.

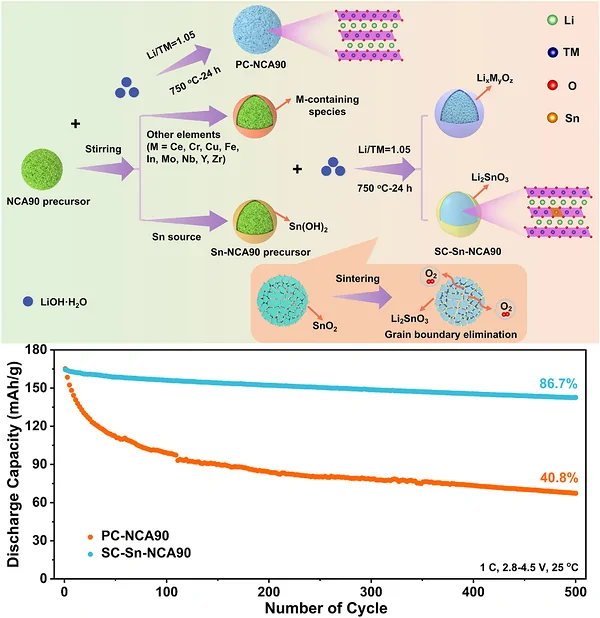

## 1 Introduction

Nickel‐rich layered lithium transition‐metal oxides are promising cathode materials for high‐energy‐density LIBs due to their high specific capacity and cost‐effectiveness, specifically those composed of Ni, Co, and Mn/Al (referred to as LiNi_x_Co_y_Mn_1‐x‐y_O_2_ or LiNi_x_Co_y_Al_1‐x‐y_O_2_, x ≥ 0.8) [1, 2, 3, 4]. Currently, commercially available nickel‐rich cathodes are polycrystalline microspheres composed of nano‐sized primary particles, suffering from significant anisotropic volume changes among adjacent primary particles and severe surface degradation during cycling, which further induces the formation of intergranular microcracks and leads to dramatic capacity fading as well as thermal runaway in LIBs [5, 6, 7]. To circumvent the inherent vulnerabilities of polycrystalline structures, single crystallization, which features a grain‐boundary‐free structure, has emerged as a compelling and highly effective strategy [8, 9, 10, 11]. However, the synthesis of single‐crystalline Ni‐rich cathodes presents substantial thermodynamic and kinetic hurdles. Achieving the requisite particle growth and complete grain boundary fusion typically demands aggressive calcination protocols, most notably elevated sintering temperature (>850°C) [12, 13], excess lithium salt [14, 15], or molten salt assistance [16, 17] to accelerate mass transport. However, elevated temperatures provoke substantial lithium volatilization and concurrent oxygen loss, triggering serious Li/Ni cation mixing that deteriorates the layered structural ordering [13, 18]. For specific NCA compositions, the situation is exacerbated: calcination above 820°C induces the segregation of electrochemically inert Li_5_AlO_4_ impurities, leading to a concurrent depletion of both Li and Al from the active bulk lattice [19]. Furthermore, excess lithium salt and molten salt assistants require a washing process to separate the impurity salts, and the clean particle surface is susceptible to side reactions, resulting in a thicker rock‐salt phase during cycling [20]. Moreover, highly soluble aluminum ions can be dissolved during the washing process, leading to structural damage [21]. Consequently, it remains an extraordinary challenge to engineer a methodology that can yield well‐dispersed single‐crystalline Ni‐rich cathodes under the mild, low‐temperature conditions characteristic of standard polycrystalline synthesis.

Furthermore, securing a single‐crystalline morphology does not entirely inoculate Ni‐rich cathodes against electrochemical degradation, particularly under the demanding conditions of high cut‐off voltages (>4.3 V vs. Li/Li^+^) required to high‐energy density. For example, Ni^4+^ ions are thermodynamically unstable and tend to be reduced to the more stable Ni^2+^ ions by electrolytes in the deeply delithiated state, thereby triggering the formation of rock‐salt phase on the surface [22, 23]. In the deeply delithiated state, the oxidation and escape of lattice oxygen occur due to the overlap between the O 2p band and the Ni/Co 3d band, providing a more thermodynamically advantageous migration pathway for Ni^2+^ from transition metal (TM) layer to the Li slab, which leads to the detrimental irreversible phase transition from layered structure to spinel and/or rock‐salt phase during cycling [24, 25, 26]. Additionally, the high nickel content intensifies the phase transition from the second hexagonal phase (H2) to the third hexagonal phase (H3), causing serious lattice shrinkage and the development of intragranular cracks at high cut‐off voltages (>4.3 V versus Li/Li^+^), which results in the formation of the rock‐salt phase due to exposure of fresh surfaces to electrolytes [27, 28]. The electrochemically inert rock‐salt phase can extend from the surface into the bulk, hindering Li^+^ transport and ultimately leading to dramatic capacity fading [29, 30].

To address these intrinsic structural instabilities, element doping has proven to be a highly effective technique for extending the high‐voltage survivability of single‐crystalline Ni‐rich cathodes [31]. Various cationic dopants, such as Mg^2+^, Y^3+^, Sb^3+^, Sr^4+^, Ti^4+^, Ta^5+^, Nb^5+^, Mo^6+^, have been investigated to stabilize the host lattice [32, 33, 34, 35, 36, 37, 38, 39]. Specifically, dopant elements with stronger metal‐oxygen bonding energy can pin the lattice oxygen and elevate the energy threshold required for oxygen evolution [40, 41, 42]. In this context, Tin (Sn) emerges as an exceptionally promising candidate. The Sn─O bond possesses a remarkable dissociation energy of 548 kJ mol^−1^, substantially exceeding those of the native transition metal bonds (391.6 kJ mol^−1^ for Ni─O bond, 368 kJ mol^−1^ for Co─O bond, 512 kJ mol^−1^ for Al─O bond, and 402 kJ mol^−1^ for Mn─O bond) [43, 44]. Meanwhlie, Sn^4+^ has the same ionic radius of 0.69 Å as Ni^2+^, facilitating its substitution within the crystal lattice [45, 46]. Therefore, Sn doping presents a scientifically sound strategy to fortify the lattice integrity of single‐crystalline cathodes at high cut‐off voltages.

In this study, we introduce a fundamentally distinct approach where Sn is utilized not merely as a conventional bulk dopant, but primarily as a highly reactive morphological modifier designed to overcome the low‐temperature synthesis bottleneck of SC‐NCA. By deploying a novel grain‐boundary‐elimination mechanism, we demonstrate that the thermal decomposition of surface‐coated Sn(OH)_2_ precursor into SnO_2_ acts as a localized chemical wedge. During the early stages of calcination, this SnO_2_ actively drives targeted oxygen release precisely at the primary particle grain boundaries. This controlled interfacial gas evolution successfully disrupts inter‐particle van der Waals forces, completely exfoliating and dispersing the primary particles into discrete single crystals at an unprecedentedly low temperature of 750°C, maintaining identical energetic conditions to its traditional polycrystalline counterpart. Beyond this transformative morphological role during synthesis, the Sn seamlessly integrates into the final structure to execute a dual structural and kinetic protective function: Sn doping deep within the bulk effectively suppresses oxygen release and improves the reversibility of the hazardous H2‐H3 phase transition, while the residual formation of a lithium‐ion conductive Li_2_SnO_3_ phase on the grain surface functionally stabilizes the cathode‐electrolyte interface. The synergy of this Sn‐driven low‐temperature morphology control and dual‐site stabilization ensures superior electrochemical kinetics and drastically reduces parasitic interface reactions, providing a comprehensive understanding for the advancement of high‐energy‐density LIBs.

## 2 Results and Discussion

### 2.1 Synthetic Mechanism

**FIGURE 1:**
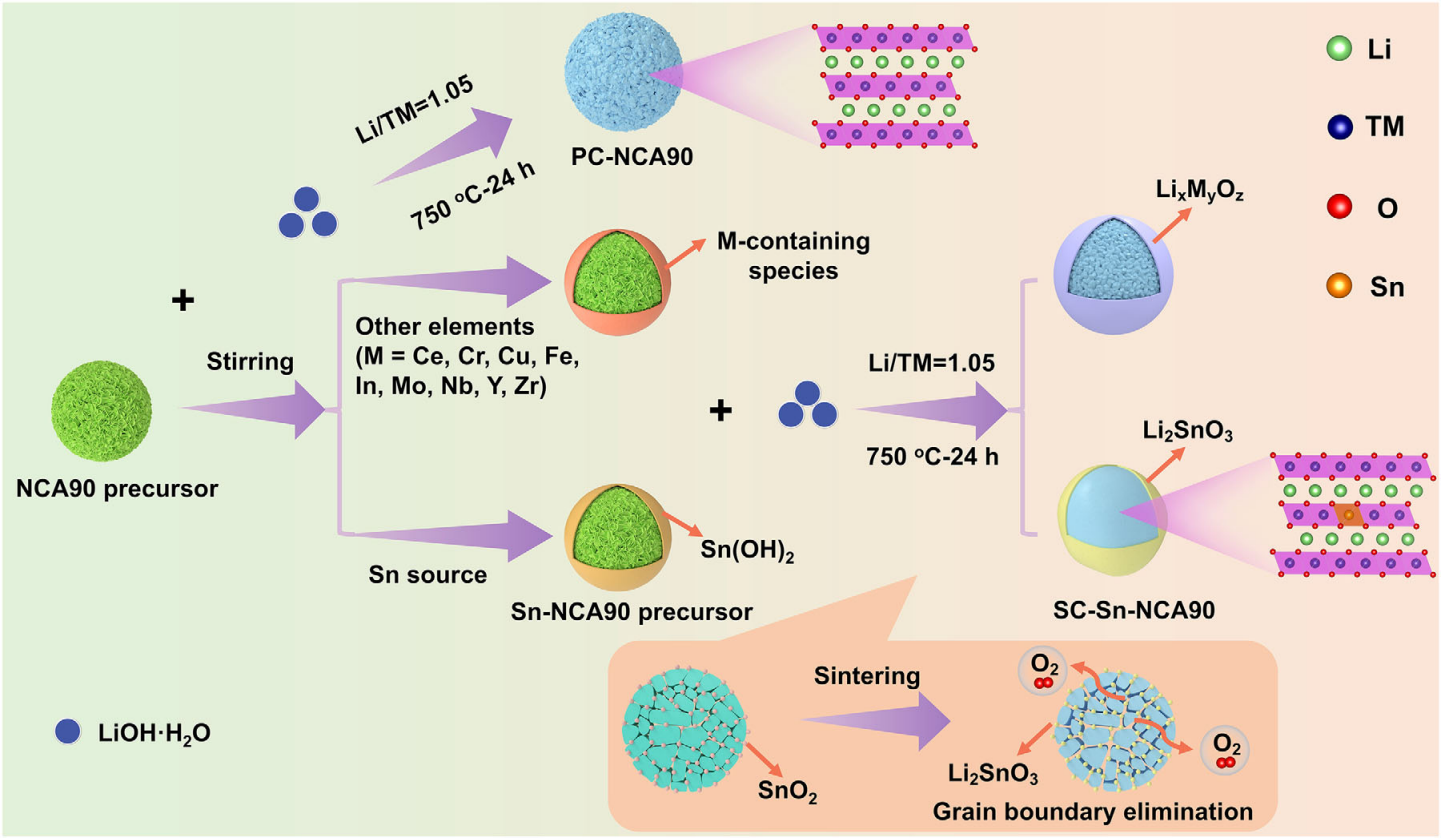
Schematic illustration of the synthesis process and crystal structure for PC‐NCA90, SC‐Sn‐NCA90 and other element‐doped polycrystalline NCA90.

Figure 1 outlines the comparative synthetic pathways and resulting crystallographic structures for both the polycrystalline counterpart (PC‐NCA90), Sn‐doped SC‐NCA (SC‐Sn‐NCA90), and other element‐doped polycrystalline NCA90. Ni_0.90_Co_0.08_Al_0.02_(OH)_2_ (labeled as NCA90 precursor) was thoroughly mixed with LiOH∙H_2_O in a molar ratio of 1:1.05 using a mortar, and the resulting mixture was calcined at 750°C for 24 h to yield the typically polycrystalline NCA (PC‐NCA). To drive single‐crystallization, an identical protocol was employed with one critical modification: the initial NCA90 precursor was uniformly coated with Sn(OH)_2_ via a controlled precipitation reaction in ethanol. Subjecting this Sn‐coated precursor to identical thermal conditions initiated the decomposition and subsequent oxidation of Sn(OH)_2_ into SnO_2_. Crucially, during the initial sintering phase to 700°C under an oxygen atmosphere, this nascent SnO_2_ preferentially localized along the primary grain boundaries. As calcination proceeded, an interfacial reaction between the localized SnO_2_ and the NCA primary particles triggered the targeted release of O_2_. This internal gas evolution physically forced the primary particles apart, sharply diminishing the cohesive van der Waals forces and initiating comprehensive grain‐boundary elimination. Consequently, highly dispersed, robust SC‐NCA crystals were successfully synthesized under significantly milder thermal conditions than conventionally possible, establishing a novel low‐temperature route for single‐crystal formation. However, unlike Sn doping, the introduction of other elements, such as Ce, Cr, Cu, Fe, In, Mo, Nb, Y, and Zr, does not contribute to the formation of single‑crystalline NCA at 750°C.

**FIGURE 2:**
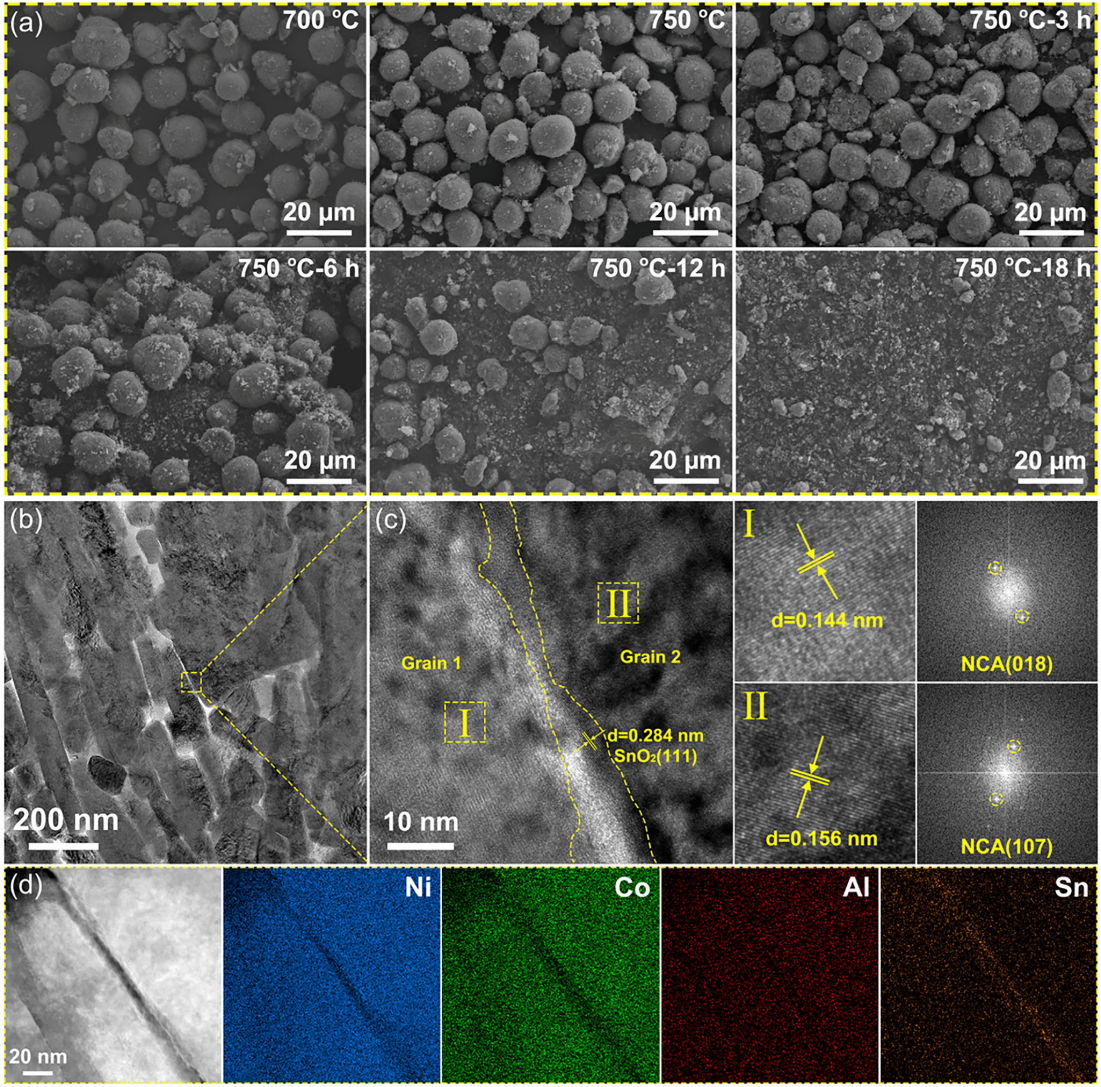
(a) SEM images of Sn‐NCA90 precursor sintered under different conditions. Cross‐sectional TEM image (b), HR‐TEM and corresponding FFT images (c) of grain boundaries for Sn‐NCA90‐700. (d) High‐magnification STEM image and the corresponding EDS elemental mappings for Sn‐NCA90‐700.

**FIGURE 3:**
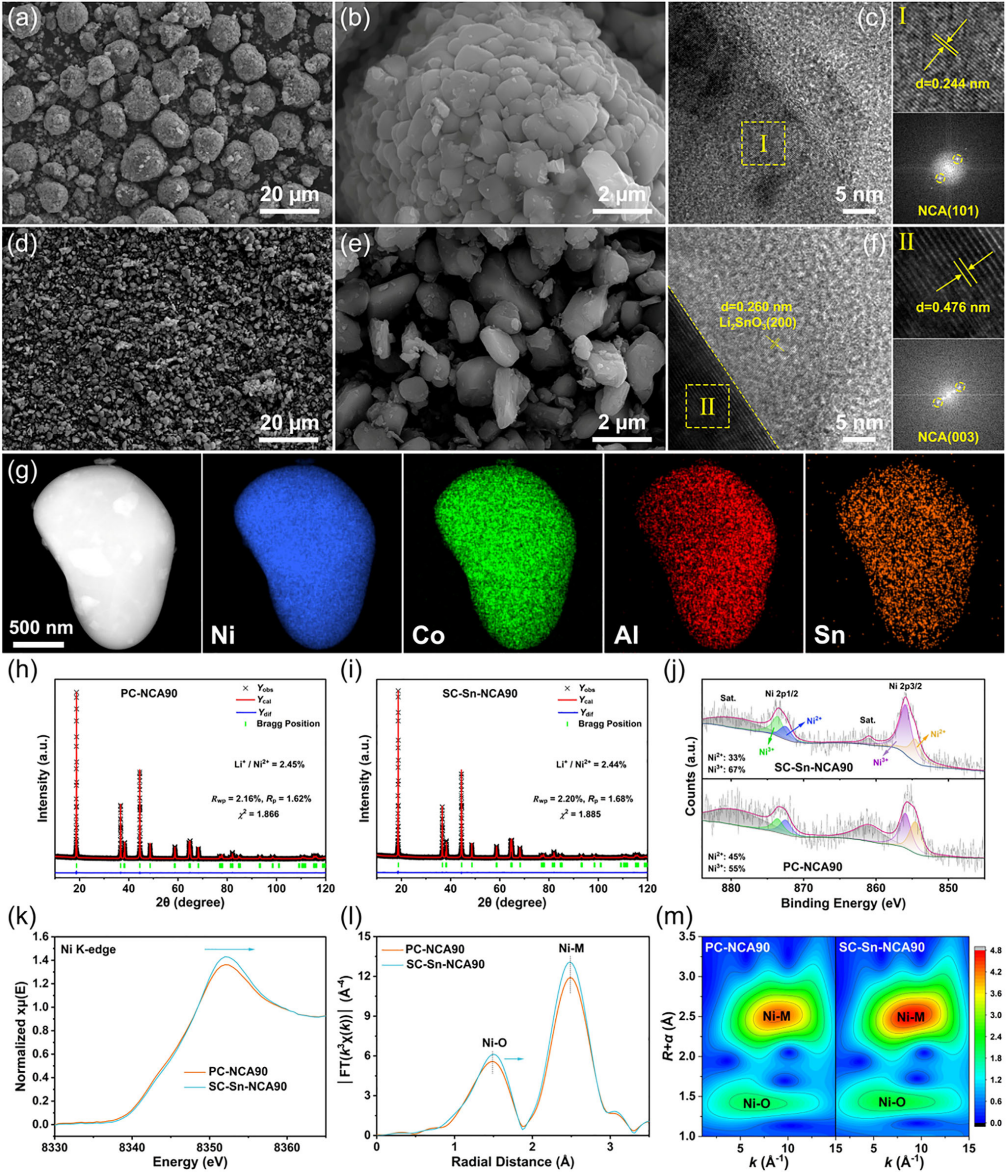
SEM images (a and b), HRTEM and corresponding FFT images (c) of PC‐NCA90. SEM images (d and e), HRTEM and corresponding images (f) of SC‐Sn‐NCA90. (g) STEM image and the corresponding EDS elemental mappings of SC‐Sn‐NCA90. Rietveld refinement results of PC‐NCA (h) and SC‐Sn‐NCA90 (i). (j) XPS spectra for Ni 2p of PC‐NCA90 and SC‐Sn‐NCA90. XANES spectra at the Ni K‐edge (k), Fourier transformation of EXAFS spectra (l) and wavelet transformed Ni K‐edge EXAFS spectra (m) for PC‐NCA90 and SC‐Sn‐NCA90.

The morphological evolution during sintering and the enrichment regions of Sn within the particles provide a compelling explanation for the single‐crystalline synthetic mechanism (Figure 2). For comparative analysis, the inherent difficulty of synthesizing SC‐NCA is clearly illustrated by evaluating the temperature‐dependent morphological evolution of the NCA90 precursor using the conventional Li/TM ratio of 1.05 (Figure S1 and Figure 3a). At the sintering temperature of 750°C, the obtained NCA (PC‐NCA90) exhibits a typical polycrystalline morphology. Elevating the calcination temperature initiates primary particle coarsening and partial fusion, but complete detachment into well‐dispersed single crystals is not realized until an excessive 900°C. Rietveld refinement of the corresponding X‐ray diffraction (XRD) patterns (Figure S2 and Figure 3h and Table S1) confirms that all intermediate and final products maintain a well‐defined hexagonal α‐NaFeO_2_ structure (*R*‐3m space group) devoid of secondary phase impurities. However, refinement also exposes a severe structural penalty intrinsic to high‐temperature processing: the degree of detrimental Li/Ni anti‐site mixing scales aggressively with temperature, surging from a manageable 2.45% at 750°C to a substantial 6.97% at 900°C. Therefore, the high sintering temperature facilitates the synthesis of SC‐NCA, but it also results in serious Li/Ni cation mixing, which exacerbates the structural stability.

The strategic introduction of the Sn(OH)_2_ coating decisively circumvents this thermodynamic and kinetic trade‐off. By functionally bypassing the thermal barrier for exfoliation, well‐dispersed SC‐NCA was successfully synthesized at just 750°C (SC‐Sn‐NCA90), representing an extraordinary 150°C reduction relative to the uncoated precursor (Figure S3 and Figure 3d,e). Extended particle growth was achievable at 800°C, yielding larger single crystals. Rietveld refinements (Figure S4 and Figure 3i, and Table S2) confirm excellent phase purity for all Sn‐modified variants, importantly revealing that the 750°C sample simultaneously achieved complete exfoliation and the absolute lowest degree of Li/Ni mixing. This structural fidelity translates directly into optimal electrochemical stability, outperforming higher‐temperature variants during prolonged cycling (Figure S5). Beyond calcination temperature, optimizing the Sn concentration is critical for controlled morphology (Figure S6 and Figure 3d). A low dosage of 0.2 mol% Sn to the M(NCA90 precursor) was insufficient, resulting in a heterogeneous matrix dominated by incomplete polycrystalline clusters. Complete, uniform single‐crystal separation was isolated precisely at 0.8 mol% Sn (SC‐Sn‐NCA90). Surprisingly, increasing the Sn loading beyond this optimal threshold proved counterproductive, precipitating the reappearance of polycrystalline aggregates. Consequently, the 0.8 mol% Sn ratio was identified as the preferred stoichiometric ratio, delivering maximized specific capacity and capacity retention (Figure S7). Ultimately, by enabling synthesis within a low‐temperature regime that precludes rampant high‐temperature Li/Ni cation mixing and severe Li/O volatilization, the SC‐Sn‐NCA90 demonstrates vastly superior electrochemical reversibility when directly compared to the conventional high‐temperature undoped SC‐NCA (Figure S8).

To demonstrate the mechanics governing this Sn‐driven grain boundary elimination, the morphological evolution was monitored dynamically over the calcination hold time (Figures 2a and 3d). Progressive isothermal aging at 750°C reveals primary particles actively peeling away from the polycrystalline matrix, culminating in fully liberated SC‐Sn‐NCA90 crystals at 24 h. XRD patterns of Sn‐NCA90 precursor undergoing the early stages of the sintering process are presented in Figure S9, indicating that the pure NCA phase emerges when the Sn‐NCA90 precursor is sintered to 700°C (denoted Sn‐NCA90‐700). X‐ray photoelectron spectroscopy (XPS) analysis of Sn‐NCA90‐700 juxtaposed with the final SC‐Sn‐NCA90 product (Figures S10 and S11) reveals a significantly lower surface Sn/Ni ratio for the early‐stage intermediate. This critical observation dictates that Sn does not simply coat the macroscopic secondary particle exterior, but actively diffuses into the deep, porous interior bulk during the initial thermal ramp. This inward migration is definitively corroborated via cross‐sectional scanning transmission electron microscopy (STEM) and corresponding energy‐dispersive X‐ray spectroscopy (EDS) mapping (Figure S12). High‐resolution TEM (HRTEM) of these boundaries resolves overlapping domains: lattice spacings of 0.144 nm and 0.156 nm correspond to the (018) and (107) facets of NCA, while an interfacial fringe of 0.284 nm identifies the (111) plane of SnO_2_ (Figure 2b,c). This crystallographic evidence conclusively proves that SnO_2_ strategically locates at the grain boundaries during the early sintering phase up to 700°C. Furthermore, the high magnification STEM image and corresponding EDS elemental mappings clearly confirm that Sn is present at the grain boundaries of Sn‐NCA90‐70 (Figure 2d). Driven by further calcination, this boundary‐localized SnO_2_ reacts with the neighboring NCA primary particles via the following reaction [41, 47]:

(1) $$\mathrm{LiM}O_{2}+\alpha \mathrm{Sn}O_{2}\to Li_{1-2\alpha }MO_{2-2\alpha }+\alpha Li_{2}\mathrm{Sn}O_{3}+\frac{\alpha }{2}O_{2}\;\left(↑\right)$$

where M═Ni, Co, Al. To grain a more thorough understanding of the processes by which SnO_2_ reacts at the grain boundaries, the reaction energies of oxygen loss and corresponding models used in density functional theory (DFT) calculations for NCA and NCA + SnO_2_ are presented in Figures S13 and S14a,b. The reaction energies of oxygen loss for NCA + SnO_2_ is calculated to be 1.51 eV, which is lower than the 2.66 eV for NCA alone, indicating that SnO_2_ can accelerate oxygen release. Furthermore, thermogravimetric (TG) analysis was carried out to quantify the release of O_2_ gas induced by SnO_2_ (Figure S15). Throughout the synthetic processes for PC‐NCA90 and SC‐Sn‐NCA90, Li/O loss starts at 675°C (Figure S15a,b). Taking the mass at 675°C as the reference, the remaining proportion mass is 98.433% for SC‐Sn‐NCA90 and 98.676% for PC‐NCA90 (Figure S15c). When excluding the Li/O loss during the sintering process, the quantified release of O_2_ gas induced by SnO_2_ is measured at 0.243%. PC‐NCA is mainly formed by the coalescence of primary particles driven by adhesion force, primarily Van der Waals force, and can be calculated using the following Equation 2 [12]:

(2) $$F_{W}=\frac{h_{w}d_{p}R_{as}}{8\pi Z_{0}^{2}}\left[1+\frac{h_{w}}{8\pi^{2}Z_{0}^{3}H\left(t\right)}\right]$$

where *h*_w_ represents the Van der Waals constant, *d*_p_ represents the average diameter of primary particles, *R*_as_ represents the surface roughness, *H*(t) represents the hardness (which is related to young's modulus), *Z*_0_ represents the shortest distance between primary particles. According to Equation 2, the Van der Waals force is significantly influenced by *Z*_0_, which is the shortest distance between primary particles. Therefore, the generation of O_2_ gas at the grain boundaries separates the primary particles and increases *Z*_0_, which leads to the decrease in adhesion force and promotes the exfoliation of single crystals. This describes the synthetic mechanism of SC‐NCA, wherein Sn doping occurs as a result of the sintering process.

The unique capability of Sn to trigger this low‐temperature mechanical exfoliation was rigorously validated against other transition metal dopants. Substituting the precursor coating with alternative species and calcining at 750°C universally failed to disrupt the agglomerates, yielding standard polycrystalline morphologies equivalent to an undoped baseline (Figure S16). Exfoliation for these variants demanded a minimum calcination temperature of 800°C (Figure S17). To interrogate this discrepancy, comparative DFT reaction energies for interfacial oxygen loss were evaluated for NCA against various metal oxides (Figure S13 and S18). While SnO_2_ features a low reaction energy, both Fe_2_O_3_ and MoO_3_ demonstrated virtually identical thermodynamic favorability toward localized oxygen release. Thus, thermodynamic reaction enthalpy cannot solely account for Sn's exclusive low‐temperature efficacy. The governing distinction lies in phase kinetics and thermal stability. Previous work establishes that classical transition metal oxides lack the requisite solid‐state diffusivity to penetrate deep into NCA grain boundaries at temperatures below 800°C [41]. In Figure S19, Sn‐PC‐NCA90 retains a typical polycrystalline morphology after sintering at 750°C, whereas it transforms into single‐crystalline particles when the sintering temperature is increased to 800°C. This result indicates that the diffusion of SnO_2_ along the grain boundaries of polycrystalline NCA is limited at 750°C, thereby hindering the grain‐boundary‐elimination process. Conversely, SnO_2_ demonstrates exceptional mobility, successfully localizing at these critical boundaries by 700°C in the NCA precursor reaction system. Furthermore, Mo and Nb variants exhibited stubborn polycrystalline retention even at 800°C (Figure S17f,g). This recalcitrance stems from competitive phase instability: operando XRD studies confirm that reactive oxides like MoO_3_ and Nb_2_O_5_ chemically react with the alkaline LiOH flux prematurely, transitioning into highly stable Li_2_MoO_4_ at 650°C and LiNbO_3_ at 450°C, respectively [48]. These premature side reactions deplete the active oxide coating before it can localize and catalyze boundary oxygen release. Therefore, the singular success of Sn‐assisted grain‐boundary elimination is rooted in a trifecta of mechanistic advantages: (1) superior solid‐state diffusivity ensuring early grain boundary occupation by 700°C; (2) robust chemical inertness against premature scavenging by the harsh LiOH flux; and (3) a highly favorable interfacial oxygen evolution reaction that generates the necessary internal pneumatic pressure to shatter inter‐particle van der Waals forces.

### 2.2 Morphologies and Structures

The morphological evolution of the NCA90 precursors was initially evaluated using Scanning electron microscopy (SEM). The pristine NCA90 precursor (Figure S20a,b), upon sintering at 750°C, transforms into a conventional polycrystalline structure (PC‐NCA90) characterized by agglomerated primary particles with distinct grain boundaries (Figure 3a,b). HRTEM of PC‐NCA90 reveals a lattice spacing of 0.244 nm, corresponding to the (101) plane of the layered NCA phase (PDF #01‐086‐3777) (Figure 3c). Conversely, the Sn‐NCA90 precursor exhibits a uniform, distinct surface coating layer, verified by EDS mapping (Figure S20c‒e). Subjecting this Sn‐coated precursor to identical 750°C calcination yields SC‐Sn‐NCA90, which boasts a well‐dispersed, fully exfoliated single‐crystalline morphology (Figure 3d,e). Electron backscatter diffraction (EBSD) cross‐sectional mapping (Figure S21) confirms the monocrystalline nature of these isolated particles via uniform intra‐particle crystallographic orientations. HRTEM of SC‐Sn‐NCA90 (Figure 3f) delineates that the lattice spacing on the surface of SC‐Sn‐NCA90 is 0.260 nm, which matches the (200) plane of Li_2_SnO_3_ (PDF #00‐031‐0761), while the internal fringe spacing of 0.476 nm is indexed to (003) plane of NCA. Furthermore, STEM‐EDS mapping and cross‐sectional point analyses (Figure 3g and Figure S22) corroborate that Sn successfully diffuses from the precursor surface into the bulk lattice during calcination. The Sn 3d XPS spectrum of SC‐Sn‐NCA90 in Figure S10b exhibits peaks at 486.2 and 494.7 eV, corresponding to Sn 3d_5/2_ and 3d_3/2_, respectively, confirming that the valence state of Sn is +4 [49, 50]. Inductively coupled plasma optical emission spectroscopy (ICP‐OES) results (Table S3) confirm the incorporation of Sn while demonstrating that the relative concentrations of Li, Ni, Co, and Al remain unaffected by the doping process.

To interpret the crystallographic impact of Sn incorporation, Rietveld refinements of the XRD patterns were performed (Figure 3h,i). The *a*‐axis parameter expands marginally from 2.871 Å to 2.874 Å upon Sn doping (Tables S1 and S2, 750°C‐sintered samples). Typically, doping with a high‐valence cation like Sn^4+^ necessitates charge compensation via increased Ni^2+^ formation, which often exacerbates Li/Ni cation mixing [51]. Remarkably, the Li/Ni mixing degree in SC‐Sn‐NCA90 is 2.44%, virtually identical to the 2.45% observed for PC‐NCA90. This suppression of anti‐site defects is attributed to the exceptionally high bond dissociation energy of the Sn─O bond, which anchors lattice oxygen during high‐temperature sintering. Consequently, this inhibition prevents the reduction of TM ion migration barriers induced by oxygen vacancies, ultimately leading to a lower degree of Li/Ni cation mixing [52, 53]. XPS was employed to analyze the oxidation state of Ni on the surfaces for PC‐NCA90 and SC‐Sn‐NCA90 (Figure 3j). The fitting results indicate that the binding energy peaks for Ni 2p_3/2_ of Ni^2+^ and Ni^3+^ peaks are observed at 854.6 and 855.9 eV, respectively, while the Ni 2p_1/2_ peaks of Ni^2+^ and Ni^3+^ peaks are located at 872.5 and 873.6 eV. These peak positions are consistent with the binding energy values reported for NiO and Ni_2_O_3_ in previous studies [54, 55, 56]. The relative proportion of the detrimental Ni^2+^ species is significantly reduced from 45% in PC‐NCA90% to 33% in SC‐Sn‐NCA90, demonstrating that Sn doping actively suppresses the spontaneous surface reduction of Ni and the subsequent formation of an electrochemically inactive NiO‐like rock‐salt phase. The influence of Sn doping on the valence state and coordination environment of Ni in Ni‐rich cathodes was further investigated using X‐ray absorption spectroscopy (XAS) at the Ni K‐edge. The X‐ray absorption near edge structure (XANES) spectrum at Ni K‐edge for SC‐Sn‐NCA displays a slight shift toward higher energy compared to that of PC‐NCA90 (Figure 3k), indicating a higher valence of Ni in the doped material, which is consistent with the results observed in the XRD Rietveld refinement and XPS analysis [57]. Furthermore, Fourier and wavelet transformation of extended X‐ray absorption fine structure (EXAFS) spectra was used to investigate the local bonding environment of Ni (Figure 3l,m). In the spectra, the two primary coordination shells in the correspond to the interactions of oxygen atoms surrounding Ni (Ni─O) and the interactions between Ni and TM (Ni–TM), respectively [58, 59]. In SC‐Sn‐NCA90, the augmented intensity of the Ni─O coordination shell suggests an attenuation of the NiO_6_ octahedral distortion [59, 60]. Furthermore, an increase in the Ni─O interatomic distance is observed, accommodating the substitution of the larger Sn^4+^ ion (0.69 Å) for Ni^3+^ (0.56 Å) [45]. This result aligns with the observed increase in the lattice parameters *c* and *V* from the XRD Rietveld refinement (Table S4). Meanwhile, the Ni–TM coordination shell exhibits increased peak intensity with negligible distance alteration, confirming that the bulk transition metal framework remains highly robust following Sn integration.

### 2.3 Electrochemical Performance

**FIGURE 4:**
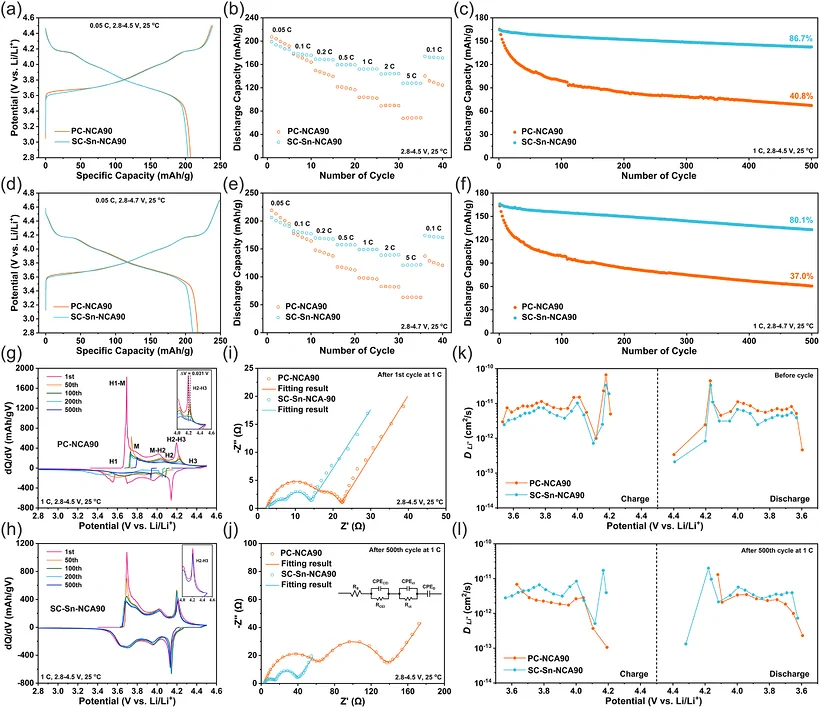
Electrochemical performance comparison of PC‐NCA90 and SC‐Sn‐NCA90 electrodes. Initial charge‐discharge at 0.05 C (a), rate capability at various discharge rates (b), and cycling stability at 1 C (c) between 2.8–4.5 V. Initial charge‐discharge at 0.05 C (d), rate capability at various discharge rates (e), and cycling stability at 1 C (f) between 2.8–4.7 V. dQ/dV curves of PC‐NCA90 (g) and SC‐Sn‐NCA90 (h) at 1 C with different cycles between 2.8–4.5 V. Nyquist plots of PC‐NCA90 and SC‐Sn‐NCA90 electrodes after first (i) and 500th (j) cycles at 1 C between 2.8–4.5 V. Li^+^ diffusion coefficient (D_Li_^+^) of PC‐NCA90 and SC‐NCA90 before (k) and after 500th (l) cycles at 1 C between 2.8–4.5 V.

To critically assess the impact of Sn doping on the electrochemical kinetics and structural longevity, the performance of PC‐NCA90 and SC‐Sn‐NCA90 cathodes was evaluated in lithium half‐cells operating between 2.8 and 4.5 V (vs Li/Li^+^). Figure 4a illustrates the initial galvanostatic charge–discharge profiles at a low current density of 0.05 C (2.8–4.5 V). The SC‐Sn‐NCA90 electrode delivers an initial charge and discharge capacity of 238.9 and 203.7 mAh g^−1^, respectively, compared to 237.3 and 207.2 mAh g^−1^ for the PC‐NCA90 baseline. The minor attenuation in initial specific discharge capacity for the single‐crystalline variant is a well‐documented phenomenon attributed to the inherent kinetic constraints of extended solid‐state lithium‐ion diffusion pathways within micron‐sized monocrystals compared to the nano‐sized primary particles of polycrystalline agglomerates [61, 62]. However, this initial kinetic limitation is comprehensively overcome at higher current densities. In Figure 4b, SC‐Sn‐NCA90 exhibits markedly superior rate capability, delivering 127.7 mAh g^−1^ at a demanding 5 C rate, nearly double the 68.1 mAh g^−1^ retained by PC‐NCA90. Most critically, the SC‐Sn‐NCA90 electrode demonstrates exceptional long‐term cycling stability (Figure 4c), maintaining a capacity retention of 86.7% after 500 cycles at 1 C, whereas the polycrystalline counterpart undergoes severe capacity decay, retaining merely 40.8%. Notably, the cycling stability of SC‐Sn‐NCA is highly competitive among recently reported single‐crystalline Ni‐rich cathodes with element doping (Figure S23). The protective efficacy of the Sn modification becomes even more pronounced under the extreme oxidative conditions of a 4.7 V cut‐off. While initial capacities at 0.05 C (2.8–4.7 V) are comparable (Figure 4d), SC‐Sn‐NCA90 dramatically outperforms PC‐NCA90 at high rates (121.2 vs. 63.3 mAh g^−1^ at 5 C, Figure 4e) and maintains an impressive 80.1% capacity retention over 500 cycles at 1 C, dwarfing the 37.0% retention of the rapidly degrading PC‐NCA90 (Figure 4f).

To reveal the fundamental structural origins of this enhanced electrochemical stability, differential capacity (dQ/dV‐V) analysis was performed to track the reversibility of phase transitions during prolonged cycling (2.8–4.5 V, 1 C). During the charging process, Ni‐rich layered oxides undergo a sequence of crystallographic phase transitions: hexagonal to monoclinic (H1‐M), monoclinic to hexagonal (M‐H2), and ultimately, a second hexagonal transition (H2‐H3) [63]. The deeply delithiated H2‐H3 transition is particularly notorious, as it precipitates a drastic and abrupt collapse of the unit cell along the *c*‐axis. This volumetric contraction generates immense localized anisotropic strain, which is the primary driver for intragranular microcracking and subsequent capacity failure [28, 64]. For the baseline PC‐NCA90 (Figure 4g), the oxidation peak corresponding to the H2‐H3 transition suffers a severe loss of intensity and a pronounced peak shift (∆V = 0.031 V) over 500 cycles. This shift reflects a catastrophic decline in phase reversibility and mounting internal polarization, diagnostic of cumulative structural degradation and electrical isolation via microcracking. Conversely, the H2‐H3 oxidation peak for the SC‐Sn‐NCA90 cathode (Figure 4h) remains remarkably resilient, displaying negligible intensity decay and minimal voltage hysteresis. This confirms that bulk Sn doping effectively pins the transition metal layers, smoothing the lattice breathing and ensuring high thermodynamic reversibility of the H2‐H3 phase transition even under continuous electrochemical cycling.

Electrochemical impedance spectroscopy (EIS) further corroborates the stabilizing influence of the surface and bulk Sn modifications. Nyquist plots (Figure 4i,j) were modeled to deconvolute the high‐frequency cathode‐electrolyte interphase resistance (R_CEI_) and the mid‐frequency charge transfer resistance (R_ct_) (fitted values in Figure S24). Following the initial formation cycle, both electrodes exhibit comparable R_ct_ values (4.36 Ω for PC‐NCA90 vs. 4.05 Ω for SC‐Sn‐NCA90). However, SC‐Sn‐NCA90 records a significantly lower initial R_CEI_ (6.83 Ω vs. 14.67 Ω), directly evidencing the beneficial role of the residual surface Li_2_SnO_3_ layer in functioning as a highly conductive, protective solid‐electrolyte interphase. The divergence becomes striking after 500 cycles: the PC‐NCA90 electrode undergoes severe impedance escalation, with R_CEI_ soaring to 61.94 Ω and R_ct_ to 65.24 Ω, indicative of unchecked parasitic side reactions and thick insulating rock‐salt phase accumulation. In stark contrast, the impedance of SC‐Sn‐NCA90 remains exceptionally stable, with R_CEI_ and R_ct_ increasing only marginally to 12.23 Ω and 23.45 Ω, respectively. Finally, galvanostatic intermittent titration technique (GITT) measurements (Figure 4k,l and Figure S25) validate the kinetic evolution. While the initial apparent Li^+^ diffusion coefficient (D_Li_+) of SC‐Sn‐NCA90 is lower than PC‐NCA90 due to the extended single‐crystal diffusion lengths [61, 62]. This dynamic completely reverses after 500 cycles. The post‐cycled SC‐Sn‐NCA90 maintains a robust D_Li_+ that significantly eclipses the structurally compromised PC‐NCA90. Together, these results unequivocally demonstrate that the dual‐site Sn modification, bulk doping to mitigate lattice strain and surface Li_2_SnO_3_ to stabilize the CEI, synergistically safeguards both the thermodynamic structural integrity and the interfacial kinetics of the Ni‐rich cathode under high‐voltage cycling.

### 2.4 Parasitic Reaction

**FIGURE 5:**
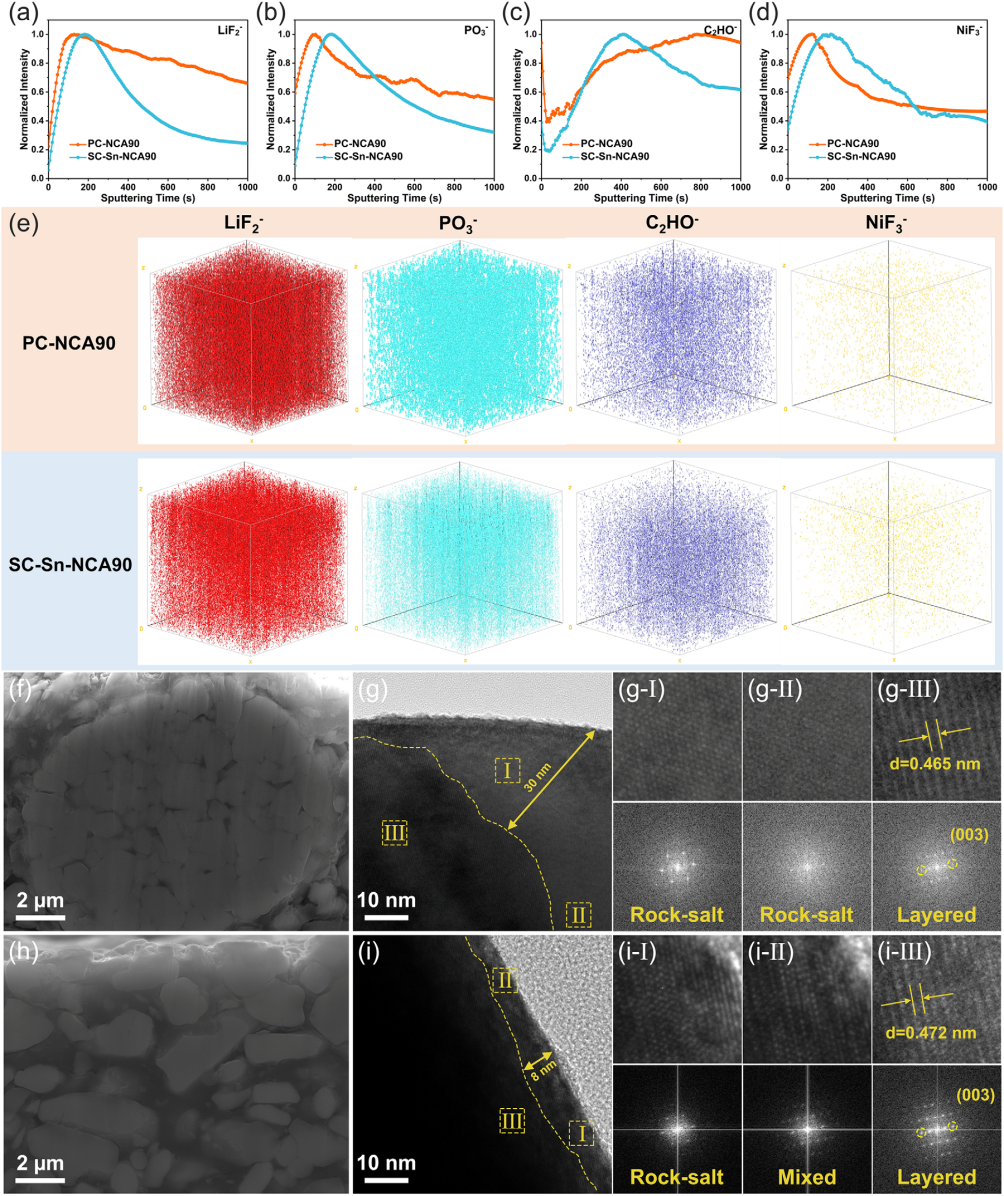
The chemical characterizations of the PC‐NCA90 and SC‐Sn‐NCA90 electrodes after 500 cycles at 1 C between 2.8–4.5 V. Normalized TOF‐SIMS depth profiles of LiF_2_^−^ (a), PO_3_^−^ (b), C_2_HO^−^ (c) and NiF_3_^−^ (d) species. (e) 3D rendering of composition (LiF_2_^−^, PO_3_^−^, C_2_HO^−^ and NiF_3_^−^) and concentration distribution. Cross‐sectional SEM images (f), HRTEM and corresponding FFT images (g) of PC‐NCA90 after 500 cycles at 1 C between 2.8–4.5 V. Cross‐sectional SEM image (h), HRTEM and corresponding images (i) of SC‐Sn‐NCA90 after 500 cycles at 1 C between 2.8–4.5 V.

To explore the influence of Sn modification on the CEI evolution during prolonged cycling, Time‐of‐Flight secondary ion mass spectrometry (TOF‐SIMS) was employed to map the compositional depth and 3D concentration distribution on the cathode surface. Organic and inorganic species such as LiF_2_^−^, PO_3_^,^ and C_2_HO^−^, which primarily originate from the oxidative decomposition of the LiPF_6_ salt and carbonate solvents, typically dominate the outer CEI layer [65]. Conversely, NiF_3_^−^ fragments, indicative of transition metal dissolution, localize within the inner CEI layer [66, 67]. After 500 cycles at 1 C (2.8–4.5 V), the normalized TOF‐SIMS depth profiles (Figure 5a–d) and corresponding 3D renderings (Figure 5e) reveal that the signal intensities of all these degradation fragments are substantially attenuated for the SC‐Sn‐NCA90 electrode compared to the PC‐NCA90 baseline. This suppressed accumulation of electrolyte decomposition products confirms that the Sn modification facilitates the formation of a thinner, more robust CEI film, which effectively mitigates parasitic interfacial reactions and minimizes electrochemical insulation, thereby sustaining efficient Li^+^ transport.

Furthermore, cross‐sectional SEM and HRTEM were utilized to evaluate the morphological and surface structural degradation after prolonged cycling. In Figure 5f, the cycled PC‐NCA90 suffers from extensive intergranular microcracking, a primary cause of electrical isolation and accelerated electrolyte penetration. HRTEM (Figure 5g) further exposes a thick, pervasive rock‐salt phase on the PC‐NCA90 surface, which severely impedes Li^+^ diffusion and drives rapid capacity decay. In striking contrast, the SC‐Sn‐NCA90 cathode maintains its macroscopic structural integrity, entirely devoid of stress‐induced intragranular fractures (Figure 5h). At the nanoscale, HRTEM (Figure 5i) demonstrates that the phase transformation in SC‐Sn‐NCA90 is highly restricted, yielding only a minimal, disordered rock‐salt/layered mixture restricted to a narrow surface layer (∼8 nm). These results unequivocally establish that Sn doping not only preserves the monolithic mechanical stability of the single crystals but also thermodynamically suppresses the destructive layered‐to‐rock‐salt phase transition at the cathode‐electrolyte boundary under high‐voltage operation.

### 2.5 Theoretical Calculations

**FIGURE 6:**
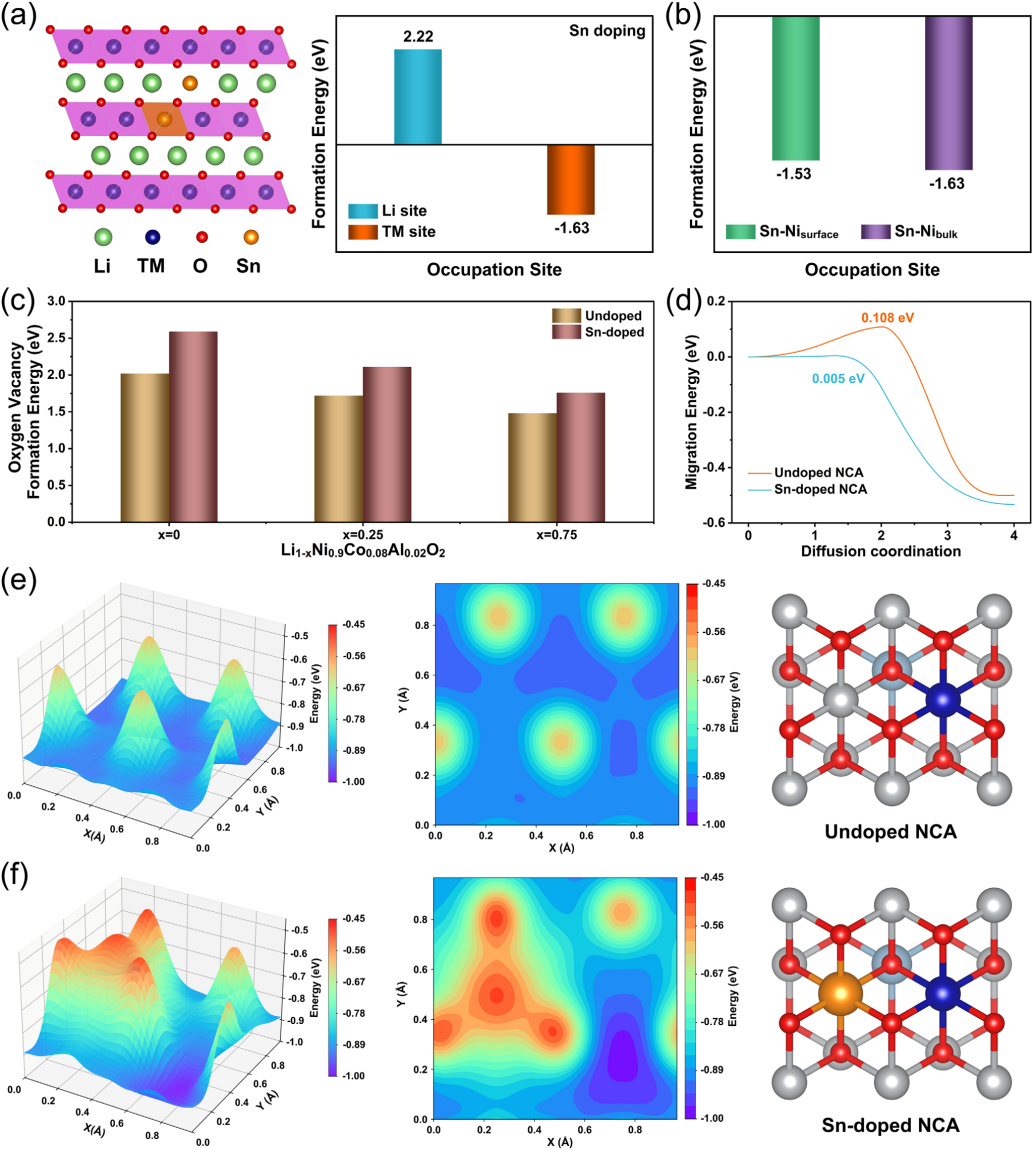
(a) Schematic diagram of the different lattice sites for Sn atoms and their corresponding energies. (b) Formation energy for substituting Ni atoms near the surface and within the bulk with Sn. (c) Oxygen vacancy formation energy for both undoped and Sn‐doped NCA under different delithiated states. (d) Li^+^ migration energy for undoped and Sn‐doped NCA. 3D/2D PES scan of Li^+^ and the corresponding models for undoped NCA (e) and Sn‐doped NCA (f).

To explain the thermodynamic origins of the enhanced structural integrity in SC‐Sn‐NCA90, density functional theory (DFT) calculations were performed (Figure 6). The formation energy for Sn substitution at the Li site is highly positive (2.22 eV), whereas substitution at the Ni site is thermodynamically favorable (−1.63 eV, Figure 6a). This confirms that Sn preferentially occupies the TM layer. Furthermore, the substitution of Ni by Sn exhibits a lower formation energy within the bulk (−1.63 eV) compared to the near‐surface region (−1.53 eV) (Figure 6b and Figure S26), indicating a thermodynamic preference for bulk integration. Because lattice oxygen oxidation and subsequent O_2_ release drive structural degradation, oxygen vacancy formation energies were evaluated across various delithiated states (Figure 6c). Sn‐doped NCA consistently exhibits oxygen vacancy formation energies than undoped NCA, demonstrating that bulk Sn doping firmly anchors the lattice oxygen. This robust oxygen retention is critical for preserving the layered framework's structural stability during high‐voltage cycling.

Kinetically, Sn doping significantly facilitates Li^+^ transport. The calculated activation energy barrier for Li^+^ migration in Sn‐doped NCA is drastically reduced to 0.006 eV, compared to 0.108 eV for the pristine material (Figure 6d and Figure S27). Additionally, potential energy surface (PES) scans (Figure 6e,f) reveal that the localized energy of Li^+^ adjacent to the Sn dopant is noticeably elevated. This elevated local energy destabilizes the resting Li^+^ state, thereby increasing its thermodynamic activity and driving force for diffusion. These theoretical insights, enhanced bulk structural stability combined with a significantly flattened Li^+^ migration barrier, perfectly corroborate the superior rate capability and cycling resilience experimentally observed for the SC‐Sn‐NCA90 cathode (Figure 4b,e).

### 2.6 Approach Extension

To extend the application of the synthetic method for SC‐NCA through Sn doping, several NCA precursors were investigated. The elemental compositions of these precursors were analyzed by ICP‐OES, and the detailed results were depicted in Table S4. Despite differences in morphology, size, and proportion of TM elements compared to the NCA90 precursor, these NCA precursors exhibit the same behavior (Figures S28–S31). At a sintering temperature of 750°C, the samples derived from these NCA precursors also display a typical polycrystalline morphology. After these NCA precursors were coated with Sn(OH)_2_, well‐dispersed SC‐NCA was successfully synthesized. Furthermore, NCA82 precursor was selected to confirm the improved electrochemical performance attributed to Sn doping (Figure S32). The obtained SC‐Sn‐NCA82 shows superior initial capacity, rate performance, and cycling stability compared to PC‐NCA82 at elevated cut‐off voltages of 4.5 and 4.7 V. These findings indicate that the effect of Sn doping on both the synthesis and improved electrochemical performance of SC‐NCA is of wide significance and highly convincing.

## 3 Conclusions

In summary, we successfully prepared the low‐temperature synthesis of single‐crystalline NCA via a novel Sn‐assisted grain‐boundary‐elimination mechanism, operating at just 750°C–150°C lower than conventional undoped methods. During initial calcination, the Sn(OH)_2_ precursor converts to SnO_2_, which localizes at primary grain boundaries. This SnO_2_ acts as a chemical wedge, driving localized O_2_ evolution that disrupts inter‐particle van der Waals forces and facilitates complete single‐crystal exfoliation. Owing to the absence of grain boundaries, the as‐obtained SC‐Sn‐NCA90 effectively suppresses intergranular microcrack formation, in contrast to PC‐NCA90 under high cut‐off voltage cycling. In addition, Sn doping within the bulk of the grain effectively stabilizes the lattice oxygen and suppresses detrimental layered‐to‐rock‐salt phase transitions, while the residual formation of a highly conductive Li_2_SnO_3_ surface layer mitigates interfacial parasitic reactions and accelerates Li^+^ diffusion kinetics at high cut‐off voltages. The synergistic effects of Sn doping and Li_2_SnO_3_ coating collectively enhance the high‐voltage stability. Consequently, the resulting SC‐Sn‐NCA90 cathode delivers exceptional rate capability and structural longevity, maintaining an impressive 86.7% capacity retention after 500 cycles at 1 C (2.8–4.5 V), vastly outperforming its polycrystalline baseline. Even under a more aggressive condition (2.8–4.7 V, 1 C, 500 cycles), it achieves 80.1% capacity retention, significantly surpassing the polycrystalline counterpart. This study offers valuable insights into the synthesis of single‐crystalline Ni‐rich layered cathode materials, including NCA, NCM, and Co‐free variants. The element‐driven elimination of grain boundaries, exemplified by Sn doping, enables the synthesis of such materials at reduced sintering temperatures. Moreover, these dopant elements themselves enhance electrochemical performance, making this a promising strategy for the future design of cathode materials with excellent capacity and cycling stability at high cut‐off voltages.

## 4 Experimental Section

### 4.1 Materials Preparation

The Ni_0.90_Co_0.08_Al_0.02_(OH)_2_ was purchased from Guangdong Fangyuan Environment Co., Ltd, which was labeled as NCA90 precursor. SnCl_2_∙H_2_O (≥98%), LiOH∙H_2_O (≥99%) and EtOH (≥99.8%) were purchased from Shanghai Aladdin Biochemical Technology Co., Ltd. For Sn(OH)_2_ coated NCA90 precursor, NCA90 precursor and LiOH·H_2_O were putted into EtOH under stirring, the molar ratio of Sn and LiOH·H_2_O was 1:4. Then, SnCl_2_∙H_2_O was dissolved in EtOH, and the desired coating level of Sn(OH)_2_ and NCA90 precursor was 0.8 mol%. SnCl_2_∙H_2_O/EtOH was added to the stirred solution and left to stir for 2 h at room temperature. The obtained Sn(OH)_2_ coated NCA90 precursor was washed with EtOH and collected by centrifugation, which was labeled as Sn‐NCA90 precursor. As‐obtained Sn‐NCA90 precursor was mixed with LiOH·H_2_O (Li/TM = 1.05) and sintered at 500°C for 5 h, followed by sintering at 750°C for 24 h, yielding the product labeled as SC‐Sn‐NCA90. Different Sn content, sintering temperature, and time were studied in‐depth to understand the effect of Sn on the synthesis of SC‐NCA. In comparison, the NCA90 precursor was directly mixed with LiOH·H_2_O (Li/TM = 1.05) and sintered at 500°C for 5 h, followed by sintering at 750°C for 24 h, resulting in the product labeled as PC‐NCA90. Various sintering temperatures were also investigated to gain insights into the synthesis of SC‐NCA without Sn.

### 4.2 Physical Characterizations

The morphological structure of the samples was characterized using field emission scanning electron microscopy (FESEM, Merlin Comact, Carl Zeiss) and high‐resolution transmission electron microscopy (TEM, FEI Tecnai G20 F20). For cross‐sectional TEM analysis, including scanning transmission electron microscopy (STEM) and high‐resolution TEM (HRTEM), the samples were prepared using a focused ion beam (FIB) system (FEI Scios 2 HiVac) and characterized with TEM (FEI Talos F200X G2). EBSD mapping was performed using a FESEM (Gemini 560, Carl Zeiss) equipped with a Symmetry S2 EBSD detector (Oxford Instruments). The step size of the EBSD maps was set at 80 nm. XRD (Panalytical X'Pert) was utilized to analyze the crystal structure of the samples in the 2*θ* range from 10° to 120° using Cu K*α* irradiation. The XRD data were refined with the EXPGUI software package [68]. XPS was conducted using the Thermo Fisher Scientific K‐Alpha system. X‐ray absorption fine structure (XAFS) spectroscopy was conducted using a Table XAFS‐3000(A) (Anhui Specreation Instrument Technology Co., Ltd.) in transmission mode at 25 kV and 20 mA. A Si (551) spherically bent crystal analyzer with a radius of curvature of 500 mm was employed to analyze nickel (Ni). XAFS spectroscopy data were analyzed with the Athena software [69]. The *k* ^3^‐weighted χ(*k*) data were Fourier transformed after the application of a Hanning window function. The wavelet transform of the EXAFS data was conducted with the Hama Fortran program [70, 71]. The cross‐sectional cathodes were polished using a cross‐section polisher (JEOL, IB‐09020CP). TOF‐SIMS was conducted with a focused ion beam scanning electron double‐beam electron microscope (FIB‐SEM, TESCAN SOLARIS) equipped with a C‐TOF. ICP‐OES (Agilent 5110) was performed to analyze the elemental content of the samples. TG analysis was carried out using a synchronous thermal analyzer (NETZSCH, STA 449 F3).

### 4.3 Electrochemical Tests

The cathode slurries were prepared by mixing the active materials, conductive carbon black (super P), conductive graphite, and poly (vinylidene fluoride) (PVDF) in a mass ratio of 90:3:2:5, using N‐methyl‐2‐pyrrolidone (NMP) as the solvent. The slurries were casted evenly on aluminum foil in a loading of ∼2.4 mg cm^−2^ with doctor blade and then vacuum dried at 120°C for 10 h to remove the solvent. The half cells were assembled in an argon‐filled glovebox, utilizing lithium metal as both the reference and counter electrode, along with a separator (Celgard M824) and an electrolyte composed of 1 M LiPF_6_ in ethylene carbonate, diethyl carbonate and dimethyl carbonate (1:1:1 wt.%) with 1 wt.% tri(trimethylsilyl)phosphite and 3 wt.% fluoroethylene carbonate as additives. After standing for 24 h, the assembled half cells were subjected to charge‐discharge tests using a battery tester (CT3002A, Wuhan Land Electronics Co., Ltd) at 25°C. The cycle performance was measured at a constant C‐rate of 0.05 C for initial three cycles, followed by 1 C for 500 cycles (1 C = 200 mA g^−1^) between 2.8–4.5 V or 4.7 V. Rate performance was evaluated at the current densities from 0.05 to 5 C between 2.8–4.5 V or 4.7 V. Electrochemical impedance spectroscopy was conducted using an electrochemical workstation (Autolab PGSTAT302) over a frequency range of 0.01 Hz to 1 MHz with an AC amplitude of 5 mV, after allowing the half cells to rest for 2 h at 50% state of charge (SOC) under open circuit conditions. The SOC was calculated using the charge capacity of the last cycle. The galvanostatic intermittent titration technique (GITT) measurements were performed with a battery tester (CT3002A) before and after 500 cycles at 1 C between 2.8–4.5 V. The half cells underwent testing at a constant current of 0.1 C between 2.8–4.5 V for 30 min.

### 4.4 Computational Details

The DFT calculations were performed with the projector augmented wave (PAW) method and Perdew–Burke–Ernzerhof (PBE) exchange‐correlation functional by Vienna ab initio simulation package (VASP) [72, 73, 74, 75]. The plane‐wave energy cutoff was set to be 450 eV. For geometry optimization, the convergence criteria for electronic energy and atomic forces were set at 10^−5^ eV and 0.01 eV/Å, respectively. The formation energy (∆*E*_form_) of Sn doping was calculated to confirm the occupation sites and positions. It is defined as: ∆E_form_ = *E*_slab‐m_ + *E*_m_^0^– *E*_slab_ – *E*_m_, where *E*_slab‐m_ and *E*_slab_ represent the total energy of slab with the metal doped and the energy of the clean slab, respectively, while *E*_m_^0^ and *E*_m_ denote the energies of the dopant atoms and host metal atoms, respectively [76]. To investigate the effects of Sn doping on the thermodynamic stability of lattice oxygen, the oxygen vacancy formation energies under various delithiated states were calculated. This is defined as: *V*_O_ = *E*(Li_x_M_y_O_2‐δ_) + $\frac{\delta }{2}$ E(O_2_)‐E(Li_x_M_y_O_2_), where *E*(Li_x_M_y_O_2‐δ_) represents the internal energy of the supercell with an oxygen vacancy, *E*(Li_x_M_y_O_2_) is the energy of the supercell without the vacancy, and *E*(O_2_) denotes the energy of oxygen [77]. The migration energy of Li^+^ was calculated using the climbing image nudged elastic band (CI‐NEB) method, with the pathways optimized until the maximum force was less than 0.02 eV/Å [78]. The PES scan of Li^+^ was conducted by calculating the insertion energy at different positions within a fixed transition metal oxide layer, using a scan matrix size of 35 × 35.

### 4.5 Declaration of Competing Interest

The authors declare that they have no known competing financial interest or personal relationships that could have appeared to influence the work reported in this paper.

## Author Contributions

**Guomeng Xie**: conceptualization, writing – original draft, investigation, formal analysis. **Delai Qian**: methodology, software, data curation, investigation, visualization. **Ruiwu Li**: investigation, validation, formal analysis, project administration, resources. **Terence Xiaoteng Liu**: writing – review and editing. **Shun Lu**: writing – review and editing, supervision, funding acquisition.

## Conflicts of Interest

The authors declare no conflicts of interest

## Supporting information




**Supporting File**: advs77769‐sup‐0001‐SuppMat.docx.

## Data Availability

The data that support the findings of this study are available from the corresponding author upon reasonable request.

## References

[1] J. Lu , C. Xu , W. Dose , et al., “Microstructures of Layered Ni‐Rich Cathodes for Lithium‐Ion Batteries,” Chemical Society Reviews 53, no. 9 (2024): 4707–4740, 10.1039/d3cs00741c. 38536022

[2] W. Li , E. M. Erickson , and A. Manthiram , “High‐nickel Layered Oxide Cathodes for Lithium‐Based Automotive Batteries,” Nature Energy 5, no. 1 (2020): 26–34, 10.1038/s41560-019-0513-0.

[3] G. Zhao , Y. Sun , H. Ma , et al., “Exploring Degradation Mechanisms and Recent Developments in High‐Nickel Layered Cathodes for Lithium Batteries,” Electrochemical Energy Reviews 8, no. 1 (2025): 21, 10.1007/s41918-025-00254-z.

[4] H. Yu , J. Cheng , H. Zhu , et al., “Reversible Configurations of 3‐Coordinate and 4‐Coordinate Boron Stabilize Ultrahigh‐Ni Cathodes With Superior Cycling Stability for Practical Li‐Ion Batteries,” Advanced Materials 37, no. 1 (2024): 2412360, 10.1002/adma.202412360. 39473297

[5] J. Kim , H. Lee , H. Cha , M. Yoon , M. Park , and J. Cho , “Prospect and Reality of Ni‐Rich Cathode for Commercialization,” Advanced Energy Materials 8, no. 6 (2018): 1702028, 10.1002/aenm.201702028.

[6] W. Li , H. Y. Asl , Q. Xie , and A. Manthiram , “Collapse of LiNi_1–x‐y_Co_x_Mn_y_O_2_ Lattice at Deep Charge Irrespective of Nickel Content in Lithium‐Ion Batteries,” Journal of the American Chemical Society 141, no. 13 (2019): 5097–5101, 10.1021/jacs.8b13798. 30887807

[7] J. Li , L. E. Downie , L. Ma , W. Qiu , and J. R. Dahn , “Study of the Failure Mechanisms of LiNi_0.8_Mn_0.1_Co_0.1_O_2_ Cathode Material for Lithium Ion Batteries,” Journal of The Electrochemical Society 162, no. 7 (2015): A1401–A1408, 10.1149/2.1011507jes.

[8] W. Zhou , H. Huang , X. Liu , et al., “Perspective on the Preparation Methods of Single Crystalline High Nickel Oxide Cathode Materials,” Advanced Energy Materials 13, no. 32 (2023): 2300378, 10.1002/aenm.202300378.

[9] M. J. W. Ogley , B. I. J. Johnston , D. S. Hall , and L. F. J. Piper , “Understanding Degradation in Single‐Crystalline Ni‐Rich Li‐Ion Battery Cathodes,” Chemical Reviews 125, no. 20 (2025): 9774–9806, 10.1021/acs.chemrev.5c00330. 41066158 PMC12550819

[10] Y. Chu , K. Wang , C. Chen , et al., “Constructing Stable Sub/Surface Structure to Boost Superior Cyclabilities of Single‐Crystalline Ni‐Rich Cathode,” Advanced Science 12, no. 44 (2025): 10817, 10.1002/advs.202510817. PMC12667453 40884236

[11] K. Liu , J. Yang , H. Wang , et al., “Achieving Fast Charging and Superior Cycling Stability Single‐Crystal Ni‐Rich Cathodes by Ultrafast Aqueous Washing,” Advanced Science 13, no. 10 (2026): 17421, 10.1002/advs.202517421. PMC12915119 41436400

[12] L. Qiu , M. Zhang , Y. Song , et al., “Origin and Regulation of Interface Fusion During Synthesis of Single‐Crystal Ni‐Rich Cathodes,” Angewandte Chemie International Edition 62, no. 12 (2023): 202300209, 10.1002/anie.202300209. 36718610

[13] Z. Xu , Z. Wang , X. Tan , et al., “Correlating Morphological and Structural Evolution With the Electrochemical Performance of Nickel‐Rich Cathode Materials: From Polycrystal to Single Crystal,” Journal of The Electrochemical Society 169, no. 9 (2022): 090520, 10.1149/1945-7111/ac8ee1.

[14] J. Duan , C. Wu , Y. Cao , et al., “Enhanced Compacting Density and Cycling Performance of Ni‐riched Electrode via Building Mono Dispersed Micron Scaled Morphology,” Journal of Alloys and Compounds 695 (2017): 91–99, 10.1016/j.jallcom.2016.10.158.

[15] Q. Guo , J. Huang , Z. Liang , et al., “The Use of a Single‐crystal Nickel‐rich Layered NCM Cathode for Excellent Cycle Performance of Lithium‐Ion Batteries,” New Journal of Chemistry 45, no. 7 (2021): 3652–3659, 10.1039/d0nj05914e.

[16] M. Yoon , Y. Dong , Y. Huang , et al., “Eutectic Salt‐Assisted Planetary Centrifugal Deagglomeration for Single‐Crystalline Cathode Synthesis,” Nature Energy 8, no. 5 (2023): 482–491, 10.1038/s41560-023-01233-8.

[17] L. Azhari , Z. Meng , Z. Yang , G. Gao , Y. Han , and Y. Wang , “Underlying Limitations Behind Impedance Rise and Capacity Fade of Single Crystalline Ni‐rich Cathodes Synthesized via a Molten‐Salt Route,” Journal of Power Sources 545 (2022): 231963, 10.1016/j.jpowsour.2022.231963.

[18] H. Huang , L. Zhang , H. Tian , et al., “Pulse High Temperature Sintering to Prepare Single‐Crystal High Nickel Oxide Cathodes With Enhanced Electrochemical Performance,” Advanced Energy Materials 13, no. 3 (2023): 2203188, 10.1002/aenm.202203188.

[19] J. Li , N. Zhang , H. Li , et al., “Impact of the Synthesis Conditions on the Performance of LiNi_x_Co_y_Al_z_O_2_ With High Ni and Low Co Content,” Journal of The Electrochemical Society 165, no. 14 (2018): A3544–A3557, 10.1149/2.0931814jes.

[20] W. Lee , S. Lee , E. Lee , et al., “Destabilization of the Surface Structure of Ni‐rich Layered Materials by Water‐washing Process,” Energy Storage Materials 44 (2022): 441–451, 10.1016/j.ensm.2021.11.006.

[21] X. Huang , J. Duan , J. He , et al., “Ions Transfer Behavior During water washing for LiNi_0.815_Co_0.15_Al_0.035_O_2_: Role of Excess Lithium,” Materials Today Energy 17 (2020): 100440, 10.1016/j.mtener.2020.100440.

[22] B. Li , G. Rousse , L. Zhang , et al., “Constructing “Li‐rich Ni‐rich” Oxide Cathodes for High‐energy‐density Li‐ion Batteries,” Energy & Environmental Science 16, no. 3 (2023): 1210–1222, 10.1039/d2ee03969a.

[23] H. Maleki Kheimeh Sari and X. Li , “Controllable Cathode–Electrolyte Interface of Li[Ni_0.8_Co_0.1_Mn_0.1_]O_2_ for Lithium Ion Batteries: A Review,” Advanced Energy Materials 9 (2019): 1901597, 10.1002/aenm.201901597.

[24] J. Chen , W. Deng , X. Gao , et al., “Demystifying the Lattice Oxygen Redox in Layered Oxide Cathode Materials of Lithium‐Ion Batteries,” ACS Nano 15, no. 4 (2021): 6061–6104, 10.1021/acsnano.1c00304. 33792291

[25] B. Li , Z. Zhuo , L. Zhang , et al., “Decoupling the Roles of Ni and Co in Anionic Redox Activity of Li‐rich NMC Cathodes,” Nature Materials 22, no. 11 (2023): 1370–1379, 10.1038/s41563-023-01679-x. 37798516

[26] C. Zeng , R. Zheng , F. Fan , et al., “Phase Compatible Surface Engineering to Boost the Cycling Stability of Single‐Crystalline Ni‐Rich Cathode for High Energy Density Lithium‐Ion Batteries,” Energy Storage Materials 72 (2024): 103788, 10.1016/j.ensm.2024.103788.

[27] Y. Liu , W. Song , A. Eldesoky , et al., “The Impact of Upper Cut‐Off Voltage on the Cycling Performance of Li‐Ion Cells With Positive Electrodes Having Various Nickel Contents,” Journal of The Electrochemical Society 169, no. 4 (2022): 040531, 10.1149/1945-7111/ac6456.

[28] N.‐Y. Park , G.‐T. Park , S.‐B. Kim , W. Jung , B.‐C. Park , and Y.‐K. Sun , “Degradation Mechanism of Ni‐Rich Cathode Materials: Focusing on Particle Interior,” ACS Energy Letters 7, no. 7 (2022): 2362–2369, 10.1021/acsenergylett.2c01272.

[29] X. Fan , X. Ou , W. Zhao , et al., “In Situ Inorganic Conductive Network Formation in High‐voltage Single‐crystal Ni‐rich Cathodes,” Nature Communications 12, no. 1 (2021): 5320, 10.1038/s41467-021-25611-6. PMC8423756 34493735

[30] Y. Huang , P. Li , H. Wei , et al., “Revealing the Correlation Between Structural Evolution and Reversible Phase Transition of Single‐Crystalline Ni‐Rich Cathode,” ACS Nano 19, no. 26 (2025): 23719–23731, 10.1021/acsnano.5c03464. 40557938

[31] J. Lu , X. Min , W. Yan , et al., “Advances in Synthesis and Optimization of Single‐crystal Ni‐rich Cathode Materials for Lithium‐ion Batteries,” Journal of Energy Storage 128 (2025): 117221, 10.1016/j.est.2025.117221.

[32] Y. Wei , B. Hu , J. Peng , et al., “Enhanced Rate Performance and Mitigated Capacity Decay of Single‐crystal LiNi_0.8_Co_0.1_Mn_0.1_O_2_ by the Synergism of Mg Doping and V_2_O_5_ Coating,” Journal of Electroanalytical Chemistry 932 (2023): 117202, 10.1016/j.jelechem.2023.117202.

[33] P. Liu , H. Wei , T. Huang , et al., “Structural Regulation Enables High Interfacial Functionality for Ni‐Rich Single‐Crystalline Cathodes,” ACS Applied Materials & Interfaces 16, no. 40 (2024): 53740–53749, 10.1021/acsami.4c10398. 39316669

[34] Z. Yang , Z. Zhao , X. Zhang , et al., “Sb‐anchoring Single‐crystal Engineering Enables Ultra‐high‐Ni Layered Oxides With High‐voltage Tolerance and Long‐cycle Stability,” Nano Energy 132 (2024): 110413, 10.1016/j.nanoen.2024.110413.

[35] Z. Tang , Z. Feng , D. Deng , et al., “Optimization of Sr‐doping Boosting the Structural Stability for Single Crystalline LiNi_0.8_Co_0.1_Mn_0.1_O_2_ Cathode to Enhance Its Electrochemical Performance at Elevated Voltage and Temperature,” Journal of Power Sources 545 (2022): 231915, 10.1016/j.jpowsour.2022.231915.

[36] X. Kong , H. Yang , Y. Zhang , et al., “Design and Mechanism Exploration of Single‐crystalline NCM811 Materials With Superior Comprehensive Performance for Li‐ion Batteries,” Chemical Engineering Journal 452 (2023): 139431, 10.1016/j.cej.2022.139431.

[37] B. Cao , H.‐T. Fang , D. Li , and Y. Chen , “Improved High‐Voltage Cycling Stability of Single‐Crystalline LiNi_0.8_Co_0.1_Mn_0.1_O_2_ Cathode by Tantalum Doping,” Journal of Materials Chemistry A 12, no. 41 (2024): 28363–28373, 10.1039/d4ta04307c.

[38] F. Guo , Y. Hu , L. Qiu , et al., “Surface Energy Alteration‐Derived Grain Size Regulation Countering Capacity Deterioration in High‐Voltage Single‐Crystal Ni‐Rich Cathodes,” Journal of Materials Chemistry A 11, no. 22 (2023): 11819–11830, 10.1039/d2ta08703k.

[39] H. Huang , H. Zhu , J. Gao , J. Wang , M. Shao , and W. Zhou , “Grain‐Growth Inhibitor With Three‐Section‐Sintering for Highly Dispersed Single‐Crystal NCM90 Cubes,” Angewandte Chemie International Edition 63 (2023): 202314457, 10.1002/anie.202314457. 38010613

[40] Z. Yang , L. Chen , H. Zhu , Y. Zhu , H. Jiang , and C. Li , “Stabilizing Surface Chemistry and Texture of Single‐crystal Ni‐rich Cathodes for Li‐Ion Batteries,” Journal of Materials Science & Technology 125 (2022): 192–197, 10.1016/j.jmst.2022.01.037.

[41] G. Xie , Z. Xu , D. Qian , et al., “Synthesis of Single‐Crystalline Ni‐Rich Cathodes for Lithium‐Ion Batteries With Superior High‐Voltage Stability by Mo/Nb Dual Doping,” Energy & Environmental Materials (2026): e70319, 10.1002/eem2.70319.

[42] H. Zhu , J. Ying , Y. Wang , et al., “A Novel Strategy of Bulk‐Surface Al/Ti Continuous Modification for Improving Structural Stability and Electrochemical Performance of Ultra‐High‐Nickel Cathode Materials,” Chemical Engineering Journal 535 (2026): 175473, 10.1016/j.cej.2026.175473.

[43] T. T. Nguyen , U.‐H. Kim , C. S. Yoon , and Y.‐K. Sun , “Enhanced Cycling Stability of Sn‐doped Li[Ni_0.90_Co_0.05_Mn_0.05_]O_2_ via Optimization of Particle Shape and Orientation,” Chemical Engineering Journal 405 (2021): 126887, 10.1016/j.cej.2020.126887.

[44] H. He , J. Dong , D. Zhang , and C. Chang , “Effect of Nb Doping on the Behavior of NCA Cathode: Enhanced Electrochemical Performances From Improved Lattice Stability towards 4.5 V Application,” Ceramics International 46, no. 15 (2020): 24564–24574, 10.1016/j.ceramint.2020.06.244.

[45] J. Jeyakumar , M. Seenivasan , Y.‐S. Wu , et al., “Sn‐Doped/Coated Ni‐Rich LiNi_0.90_Co_0.04_Mn_0.03_Al_0.03_O_2_ Cathode Materials for Improved Electrochemical Performance of Li‐Ion Batteries,” ACS Applied Energy Materials 7, no. 11 (2024): 4919–4934, 10.1021/acsaem.4c00720.

[46] H. Zhu , R. Shen , Y. Tang , et al., “Sn‐Doping and Li_2_SnO_3_ Nano‐Coating Layer Co‐Modified LiNi_0.5_Co_0.2_Mn_0.3_O_2_ With Improved Cycle Stability at 4.6 V Cut‐off Voltage,” Nanomaterials 10, no. 5 (2020): 868, 10.3390/nano10050868. 32365929 PMC7279306

[47] Z. Zhu , D. Yu , Y. Yang , et al., “Gradient Li‐rich Oxide Cathode Particles Immunized Against Oxygen Release by a Molten Salt Treatment,” Nature Energy 4, no. 12 (2019): 1049–1058, 10.1038/s41560-019-0508-x.

[48] N.‐Y. Park , S.‐B. Kim , M.‐C. Kim , et al., “Mechanism of Doping With High‐Valence Elements for Developing Ni‐Rich Cathode Materials,” Advanced Energy Materials 13, no. 34 (2023): 202301530, 10.1002/aenm.202301530.

[49] G. Hu , M. Zhang , L. Wu , Z. Peng , K. Du , and Y. Cao , “Enhanced Electrochemical Performance of LiNi_0.5_Co_0.2_Mn_0.3_O_2_ Cathodes Produced via Nanoscale Coating of Li^+^‐Conductive Li_2_SnO_3_ ,” Electrochimica Acta 213 (2016): 547–556, 10.1016/j.electacta.2016.07.154.

[50] H. Sun , J. Wang , Q. Liu , et al., “Ag–Sn Dual‐Modified LiNi_0.8_Co_0.1_Mn_0.1_O_2_ as Cathode for Lithium Storage,” Journal of Alloys and Compounds 850 (2021): 156763, 10.1016/j.jallcom.2020.156763.

[51] P. Zhao , L. Chen , L. Yao , et al., “Compositional Gradient Design and Microstructural Engineering Enable Ultra‐Stable Quinary Ni‐Rich Cathodes in High‐Voltage Pouch Cells,” Science Bulletin 71, no. 5 (2026): 1113–1121, 10.1016/j.scib.2025.12.007. 41558939

[52] F. Kong , C. Liang , L. Wang , et al., “Kinetic Stability of Bulk LiNiO_2_ and Surface Degradation by Oxygen Evolution in LiNiO_2_ ‐Based Cathode Materials,” Advanced Energy Materials 9, no. 2 (2018): 1802586, 10.1002/aenm.201802586.

[53] S. Li , Y. Li , Z. Zhang , et al., “Interplays Between TM Migration, Cation Mixing and Oxygen Defects and Their Impacts on Degradation of Layer‐structured Oxide Cathode Materials,” Energy Storage Materials 79 (2025): 104337, 10.1016/j.ensm.2025.104337.

[54] C. P. Li , A. Proctor , and D. M. Hercules , “Curve Fitting Analysis of ESCA Ni 2p Spectra of Nickel‐Oxygen Compounds and Ni/Al_2_O_3_ Catalysts,” Applied Spectroscopy 38, no. 6 (1984): 880–886, 10.1366/0003702844554530.

[55] R. B. Shalvoy , P. J. Reucroft , and B. H. Davis , “Characterization of Coprecipitated Nickel on Silica Methanation Catalysts by X‐ray Photoelectron Spectroscopy,” Journal of Catalysis 56, no. 3 (1979): 336–348, 10.1016/0021-9517(79)90126-X.

[56] K. S. Kim and N. Winograd , “X‐ray Photoelectron Spectroscopic Studies of Nickel‐Oxygen Surfaces Using Oxygen and Argon Ion‐Bombardment,” Surface Science 43, no. 2 (1974): 625–643, 10.1016/0039-6028(74)90281-7.

[57] J. Jian , S. Lin , G. Han , et al., “Mitigating Kinetic Hindrance of Single‐crystal Ni‐Rich Cathodes Through Morphology Modulation, Nickel Reduction, and Lithium Vacancy Generation Achieved by Terbium Doping,” Journal of Energy Chemistry 97 (2024): 566–574, 10.1016/j.jechem.2024.06.015.

[58] Z. Huang , J. Yan , Z. Liu , et al., “Achieving Long‐Life Ni‐Rich Cathodes With Improved Mechanical‐Chemical Properties Via Concentration Gradient Structure,” Advanced Functional Materials 34, no. 34 (2024): 2400956, 10.1002/adfm.202400956.

[59] Y. Wang , C. Chen , S. Weng , C. Li , Y. Xia , and X. Lu , “Stabilizing Ultra‐High Ni Layered Cathodes for High‐Voltage Operation Through Al Doping With Synergistic Sn Modification,” Chemical Engineering Journal 514 (2025): 163172, 10.1016/j.cej.2025.163172.

[60] B. Meng , C. Yi , J. Wang , et al., “Charge Transfer‐Driven Interfacial Bonding Boosts Cyclability of Al_2_O_3_‐Coated NCM523 for Lithium‐Ion Batteries,” Inorganic Chemistry 65, no. 31 (2026): 18423–18438, 10.1021/acs.inorgchem.6c03122. 42573372

[61] X. Deng , R. Zhang , K. Zhou , et al., “A Comparative Investigation of Single Crystal and Polycrystalline Ni‐Rich NCMs as Cathodes for Lithium‐Ion Batteries,” Energy & Environmental Materials 6, no. 3 (2022): 12331, 10.1002/eem2.12331.

[62] J. Wang , J. Huang , W. Huang , et al., “Understanding the Performance Gap Between Polycrystalline and Single‐Crystal Nickel‐Rich Layered Oxide Cathodes,” Journal of the American Chemical Society 148, no. 9 (2026): 9421–9432, 10.1021/jacs.5c18922. 41759082

[63] C. Xu , P. J. Reeves , Q. Jacquet , and C. P. Grey , “Phase Behavior During Electrochemical Cycling of Ni‐Rich Cathode Materials for Li‐Ion Batteries,” Advanced Energy Materials 11 (2020): 2003404, 10.1002/aenm.202003404.

[64] B. Namkoong , N.‐Y. Park , G.‐T. Park , et al., “High‐Energy Ni‐Rich Cathode Materials for Long‐Range and Long‐Life Electric Vehicles,” Advanced Energy Materials 12, no. 21 (2022): 2200615, 10.1002/aenm.202200615.

[65] H. Yu , Z. Ren , Z. Wang , et al., “Trace‐Cobalt Surface Engineering of Ni‐Rich Co‐Free Cathodes Unlocks High‐Power Density and Long‐Cycle Life in Pouch‐Type Li‐Ion Batteries,” ACS Nano 19, no. 42 (2025): 37314–37323, 10.1021/acsnano.5c12594. 41100839

[66] H. Bai , K. Yuan , C. Zhang , et al., “Advantageous Surface Engineering to Boost Single‐Crystal Quaternary Cathodes for High‐Energy‐Density Lithium‐Ion Batteries,” Energy Storage Materials 61 (2023): 102879, 10.1016/j.ensm.2023.102879.

[67] J. Tian , G. Wang , W. Zeng , et al., “A Bimetal Strategy for Suppressing Oxygen Release of 4.6 V High‐Voltage Single‐Crystal High‐Nickel Cathode,” Energy Storage Materials 68 (2024): 103344, 10.1016/j.ensm.2024.103344.

[68] B. H. Toby , “EXPGUI, a Graphical User Interface for GSAS,” Journal of Applied Crystallography 34, no. 2 (2001): 210–213, 10.1107/S0021889801002242.

[69] B. Ravel and M. Newville , “ATHENA, ARTEMIS, HEPHAESTUS: Data Analysis for X‐Ray Absorption Spectroscopy Using IFEFFIT,” Journal of Synchrotron Radiation 12, no. 4 (2005): 537–541, 10.1107/S0909049505012719. 15968136

[70] H. Funke , M. Chukalina , and A. C. Scheinost , “A newFEFF‐Based Wavelet for EXAFS Data Analysis,” Journal of Synchrotron Radiation 14, no. 5 (2007): 426–432, 10.1107/s0909049507031901. 17717385

[71] H. Funke , A. C. Scheinost , and M. Chukalina , “Wavelet Analysis of Extended X‐ray Absorption Fine Structure Data,” Physical Review B 71, no. 9 (2005): 094110, 10.1103/PhysRevB.71.094110.

[72] G. Kresse and J. Hafner , “Ab Initiomolecular Dynamics for Liquid Metals,” Physical Review B 47, no. 1 (1993): 558–561, 10.1103/PhysRevB.47.558. 10004490

[73] G. Kresse and J. Furthmüller , “Efficiency of Ab‐initio Total Energy Calculations for Metals and Semiconductors Using a Plane‐Wave Basis Set,” Computational Materials Science 6, no. 1 (1996): 15–50, 10.1016/0927-0256(96)00008-0. 9984901

[74] P. E. Blöchl , “Projector Augmented‐Wave Method,” Physical Review B 50 (1994): 17953–17979, 10.1103/PhysRevB.50.17953. 9976227

[75] J. P. Perdew , K. Burke , and M. Ernzerhof , “Generalized Gradient Approximation Made Simple,” Physical Review Letters 77, no. 18 (1996): 3865–3868, 10.1103/PhysRevLett.77.3865. 10062328

[76] X. Ou , T. Liu , W. Zhong , et al., “Enabling High Energy Lithium Metal Batteries via Single‐Crystal Ni‐Rich Cathode Material Co‐Doping Strategy,” Nature Communications 13, no. 1 (2022): 2319, 10.1038/s41467-022-30020-4. PMC9050889 35484128

[77] Z. Xu , X. Chen , W. Fan , et al., “High‐Entropy Rock‐Salt Surface Layer Stabilizes the Ultrahigh‐Ni Single‐Crystal Cathode,” ACS Nano 18, no. 49 (2024): 33706–33717, 10.1021/acsnano.4c13911. 39605189

[78] G. Henkelman , B. P. Uberuaga , and H. Jónsson , “A Climbing Image Nudged Elastic Band Method for Finding Saddle Points and Minimum Energy Paths,” The Journal of Chemical Physics 113, no. 22 (2000): 9901–9904, 10.1063/1.1329672.

