# Supplementary material for: Sn‐Driven Grain Boundary Elimination for Single‐Crystalline Ni‐Rich Cathodes with Superior High‐Voltage Stability in Lithium‐Ion Batteries
Source: Adv Sci (Weinh). 2026 Sep 16:e77769. Online ahead of print. doi: 10.1002/advs.77769 (PMC13583227; doi:10.1002/advs.77769)
Supplement: Supplementary file 1 [file ADVS-9999-e77769-s001.docx]

**Supporting Information**

**Sn-Driven Grain Boundary Elimination for Single-Crystalline Ni-Rich Cathodes with Superior High-Voltage Stability in Lithium-Ion Batteries**

Guomeng Xie ^a, *^, Delai Qian ^a^, Ruiwu Li ^a^ , Terence Xiaoteng Liu ^b, *^, Shun Lu ^c, *^

*a* *School of Materials Science and Engineering, Harbin Institute of Technology, Harbin 150001, China*

*b School of Engineering, Physics and Mathematics, Faculty of Science and Environment, Northumbria University, Newcastle upon Tyne, UK*

*c Chongqing Institute of Green and Intelligent Technology, Chinese Academy of Sciences, Chongqing 400714, China*

* Corresponding authors

E-mail address:

xieguomeng@foxmail.com (G. Xie)

[terence.liu@northumbria.ac.uk](mailto:terence.liu@northumbria.ac.uk) (T. Liu)

lushun@cigit.ac.cn and [shun.lu@ieee.org](mailto:shun.lu@ieee.org) (S. Lu)

Contents

Figure S1. SEM images of NCA90 precursor sintered at (a) 800 ^o^C, (b) 850 ^o^C and (c) 900 ^o^C for 24 h.	6

Figure S2. Rietveld refinement results of NCA90 precursor sintered at (a) 800 ^o^C, (b) 850 ^o^C and (c) 900 ^o^C for 24 h.	6

Figure S3. SEM images of Sn-NCA90 precursor sintered at 700 ^o^C (a and b) and 800 ^o^C (c and d) for 24 h.	6

Figure S4. Rietveld refinement results of Sn-NCA90 precursor sintered at (a) 700 ^o^C and (b) 800 ^o^C for 24 h.	7

Figure S5. Cycling stability at 1 C between 2.8–4.5 V of Sn-NCA90 precursor sintered at various sintering temperatures for 24 h.	7

Figure S6. SEM images of Sn(OH)_2_ coated NCA90 precursor with different designed ration of M(Sn) and M(NCA90 precursor) sintered at 750 ^o^C for 24 h: (a) 0.2%; (b) 0.4%; (c) 1.2%; (d) 1.6%.	8

Figure S7. Cycling stability of Sn(OH)_2_ coated NCA90 precursor with different designed ration of M(Sn) and M(NCA90 precursor) sintered at 750 ^o^C for 24 h at 1 C between 2.8–4.5 V.	8

Figure S8. Comparison of cycling stability between NCA90 precursor sintered at 900 ^o^C and SC-Sn-NCA90.	9

Figure S9. XRD patterns of Sn-NCA90 precursor undergoing the early stages of the sintering process.	10

Figure S10. XPS spectra for Sn-NCA90-700 and SC-Sn-NCA90: (a) Ni 2p; (b) Sn 3d.	10

Figure S11. Area rations of Sn/Ni in the XPS spectra for Sn-NCA90-700 and SC-Sn-NCA90.	11

Figure S12. Low magnification STEM image and the corresponding EDS elemental mappings for Sn-NCA90-700.	11

Figure S13. Reaction energies of oxygen loss for NCA, and NCA with various metal oxides.	12

Figure S14. The detail models used to evaluate the reaction energy of oxygen loss for NCA (a), NCA + SnO_2_ (b), NCA + CeO_2_ (c), NCA + Cr_2_O_3_ (d), NCA + CuO (e) and NCA + Fe_2_O_3_ (f) in DFT calculations. The crystal structures of these metal oxides and their corresponding lithium metal oxides refer to the reported literatures [1-7].	13

Figure S15. TG analysis of the synthetic processes for PC-NCA90 (a) and SC-Sn-NCA90 (b). (c) Comparison of TG results for PC-Sn-NCA and SC-Sn-NCA90 starting at 675 ^o^C.	14

Figure S16. SEM images of NCA90 precursor with various doping elements sintered at 750 ^o^C for 24 h.	14

Figure S17. SEM images of NCA90 precursor with various doping elements sintered at 800 ^o^C for 24 h.	15

Figure S19. SEM images (a and b) and the corresponding EDS mapping (c) of Sn-PC-NCA90. SEM images of Sn-PC-NCA90 sintered at 750 ^o^C (d) and 800 ^o^C (e) for 24 h.	17

Figure S20. SEM images of NCA90 precursor (a and b) and Sn-NCA90 precursor (c and d). (e) EDS mapping of Sn-NCA90 precursor.	18

Figure S21. Cross-sectional SEM image (a), the band contrast EBSD map (b) and the EBSD orientation map (c) of SC-Sn-NCA90. The three Euler angles Փ1, Փ and Փ2 denote a series of rotation about the z, x and rotated z axes, respectively. Each color corresponds to a specific crystal orientation of the grains.	18

Figure S22. (a) Cross-sectional SEM image and (b) the contents of Sn in various regions of the SC-Sn-NCA90 particle conducted by EDS point analysis (the elements included in the statistics are Ni, Co, Al, O and Sn).	19

Figure S23. Comparison of the cycling performance between this work and recently reported single-crystalline Ni-rich cathodes with element doping [15-34].	19

Figure S24. Fitting results of R_CEI_ and R_ct_ after 1st and 500th cycles at 1 C for PC-NCA90 and SC-Sn-NCA90.	20

Figure S25. GITT profiles of PC-NCA90 and SC-Sn-NCA90 before (a) and after 500th (b) cycle at 1 C between 2.8–4.5 V.	20

Figure S26. The detail models of Sn doping in the different position employed in DFT calculations.	21

Figure S27. The Li^+^ migration pathways for undoped NCA and Sn-doped NCA.	21

Figure S28. SEM images of NCA82 precursor (a and b) and Sn-NCA82 precursor (c and d). (e) EDS mapping of Sn-NCA82 precursor. SEM images of PC-NCA82 (f and g) and SC-Sn-NCA82 (h and i).	22

Figure S29. SEM images of NCA83 precursor (a and b) and Sn-NCA83 precursor (c and d). (e) EDS mapping of Sn-NCA83 precursor. SEM images of PC-NCA83 (f and g) and SC-Sn-NCA83 (h and i).	22

Figure S30. SEM images of NCA90-2 precursor (a and b) and Sn-NCA90-2 precursor (c and d). (e) EDS mapping of Sn-NCA90-2 precursor. SEM images of PC-NCA90-2 (f and g) and SC-Sn-NCA90-2 (h and i).	23

Figure S31. SEM images of NCA91 precursor (a and b) and Sn-NCA91 precursor (c and d). (e) EDS mapping of Sn-NCA91 precursor. SEM images of PC-NCA91 (f and g) and SC-Sn-NCA91 (h and i).	23

Figure S32. Electrochemical performance comparison of PC-NCA82 and SC-Sn-NCA82 cathodes. Initial charge-discharge at 0.05 C (a), rate performance (b), and cycling stability at 1 C (c) between 2.8–4.5 V. Initial charge-discharge at 0.05 C (d), rate performance (e), and cycling stability at 1 C (f) between 2.8–4.7 V.	24

Table S1. Lattice parameters of NCA90 precursor sintered at various temperatures for 24 h by Rietveld refinement.	25

Table S2. Lattice parameters of Sn-NCA90 precursor sintered at various temperatures for 24 h.	25

Table S3. ICP-OES analysis of NCA90 precursor, PC-NCA90 and SC-Sn-NCA90.	25

Table S4. ICP-OES analysis of various NCA precursor.	26

Supplementary References	27

**Materials preparation**

Some doping elements were investigated to better understand the synthetic mechanism of single-crystalline NCA (SC-NCA). CeCl_3_∙7H_2_O (≥99.99%), CrCl_3_∙6H_2_O (≥98%), Cu(NO_3_)_2_∙3H_2_O (≥99%), Fe(NO_3_)_3_∙9H_2_O (≥98%), InCl_3_∙4H_2_O (99.9%), MoCl_5_ (99.6%), NbCl_5_ (≥99%), YCl_3_∙6H_2_O (≥99.9% and ZrCl_3_ (≥99.9%) were purchased from Shanghai Aladdin Biochemical Technology Co., Ltd. The synthetic process was similar to that of Sn doping, where the desired doping level of the elements and NCA90 precursor was set at 0.8 mol%. The LiOH·H_2_O content was maintained at twice the stoichiometric amount required for complete precipitation of the dopant precursors. For instance, the molar ratio of CeCl_3_·7H_2_O to LiOH·H_2_O was set at 1:6, which is twice the theoretical ratio of 1:3 needed for Ce(OH)_3_ formation. This excess of LiOH was deliberately introduced to drive the precipitation reaction to completion.

To extend the application of the synthetic method for SC-NCA by Sn, several NCA precursors were investigated, including Ni_0.82_Co_0.16_Al_0.02_(OH)_2_ (labeled as NCA82 precursor, Hubei Wanrun New Energy Technology Co., Ltd), Ni_0.83_Co_0.11_Al_0.06_(OH)_2_ (labeled as NCA83 precursor, Ningbo Lanli New Energy Technology Co., Ltd), Ni_0.90_Co_0.06_Al_0.04_(OH)_2_ (labeled as NCA90-2 precursor, Shenzhen Youyan Technology Co., Ltd) and Ni_0.91_Co_0.04_Al_0.05_(OH)_2_ (labeled as NCA91 precursor, Guangdong Brunp Recycling Technology Co., Ltd). The synthetic process was similar to that of Ni_0.90_Co_0.08_Al_0.02_(OH)_2_ (NCA90 precursor), with the products synthesized with Sn doping labeled as SC-Sn-NCA82, SC-Sn-NCA83, SC-Sn-NCA90-2 and SC-Sn-NCA91; the products synthesized without Sn doping were labeled as PC-NCA82, PC-NCA83, PC-NCA90-2 and PC-NCA91.

To evaluate the grain‑boundary diffusion ability of SnO_2_, the obtained PC‑NCA90 (polycrystalline NCA) was coated with Sn(OH)_2_ using a synthetic route analogous to that employed for the Sn(OH)_2_‑coated NCA90 precursor, yielding the product labeled as Sn-PC-NCA90. The resultant Sn‑PC‑NCA90 was subsequently sintered at 750 °C and 800 °C for 24 h, respectively.

### **Figure S1.** SEM images of NCA90 precursor sintered at (a) 800 ^o^C, (b) 850 ^o^C and (c) 900 ^o^C for 24 h.

### **Figure S2.** Rietveld refinement results of NCA90 precursor sintered at (a) 800 ^o^C, (b) 850 ^o^C and (c) 900 ^o^C for 24 h.

### **Figure S3.** SEM images of Sn-NCA90 precursor sintered at 700 ^o^C (a and b) and 800 ^o^C (c and d) for 24 h.

### **Figure S4.** Rietveld refinement results of Sn-NCA90 precursor sintered at (a) 700 ^o^C and (b) 800 ^o^C for 24 h.

### **Figure S5.** Cycling stability at 1 C between 2.8–4.5 V of Sn-NCA90 precursor sintered at various sintering temperatures for 24 h.

### **Figure S6.** SEM images of Sn(OH)_2_ coated NCA90 precursor with different designed ration of M(Sn) and M(NCA90 precursor) sintered at 750 ^o^C for 24 h: (a) 0.2%; (b) 0.4%; (c) 1.2%; (d) 1.6%.

**Figure S7.** Cycling stability of Sn(OH)_2_ coated NCA90 precursor with different designed ration of M(Sn) and M(NCA90 precursor) sintered at 750 ^o^C for 24 h at 1 C between 2.8–4.5 V.

### **Figure S8.** Comparison of cycling stability between NCA90 precursor sintered at 900 ^o^C and SC-Sn-NCA90.

### **Figure S9.** XRD patterns of Sn-NCA90 precursor undergoing the early stages of the sintering process.

### **Figure S10.** XPS spectra for Sn-NCA90-700 and SC-Sn-NCA90: (a) Ni 2p; (b) Sn 3d.

### **Figure S11.** Area rations of Sn/Ni in the XPS spectra for Sn-NCA90-700 and SC-Sn-NCA90.

### **Figure S12.** Low magnification STEM image and the corresponding EDS elemental mappings for Sn-NCA90-700.

### **Figure S13.** Reaction energies of oxygen loss for NCA, and NCA with various metal oxides.

### **Figure S14.** The detail models used to evaluate the reaction energy of oxygen loss for NCA (a), NCA + SnO_2_ (b), NCA + CeO_2_ (c), NCA + Cr_2_O_3_ (d), NCA + CuO (e) and NCA + Fe_2_O_3_ (f) in DFT calculations. The crystal structures of these metal oxides and their corresponding lithium metal oxides refer to the reported literatures [1-7].

### **Figure S15.** TG analysis of the synthetic processes for PC-NCA90 (a) and SC-Sn-NCA90 (b). (c) Comparison of TG results for PC-Sn-NCA and SC-Sn-NCA90 starting at 675 ^o^C.

### **Figure S16.** SEM images of NCA90 precursor with various doping elements sintered at 750 ^o^C for 24 h.

### **Figure S17.** SEM images of NCA90 precursor with various doping elements sintered at 800 ^o^C for 24 h.

**Figure S18.** The detail models used to evaluate the reaction energy of oxygen loss for NCA + In_2_O_3_ (a), NCA + MoO_3_ (b), NCA + Nb_2_O_5_ (c), NCA + Y_2_O_3_ (d) and NCA + ZrO_2_ (e) in DFT calculations. The crystal structures of these metal oxides and their corresponding lithium metal oxides refer to the reported literatures [8-14].

### **Figure S19.** SEM images (a and b) and the corresponding EDS mapping (c) of Sn-PC-NCA90. SEM images of Sn-PC-NCA90 sintered at 750 ^o^C (d) and 800 ^o^C (e) for 24 h.

### **Figure S20.** SEM images of NCA90 precursor (a and b) and Sn-NCA90 precursor (c and d). (e) EDS mapping of Sn-NCA90 precursor.

### **Figure S21.** Cross-sectional SEM image (a), the band contrast EBSD map (b) and the EBSD orientation map (c) of SC-Sn-NCA90. The three Euler angles Փ1, Փ and Փ2 denote a series of rotation about the z, x and rotated z axes, respectively. Each color corresponds to a specific crystal orientation of the grains.

**Figure S22.** (a) Cross-sectional SEM image and (b) the contents of Sn in various regions of the SC-Sn-NCA90 particle conducted by EDS point analysis (the elements included in the statistics are Ni, Co, Al, O and Sn).

**Figure S23.** Comparison of the cycling performance between this work and recently reported single-crystalline Ni-rich cathodes with element doping [15-34].

**Figure S24.** Fitting results of R_CEI_ and R_ct_ after 1st and 500th cycles at 1 C for PC-NCA90 and SC-Sn-NCA90.

**Figure S25.** GITT profiles of PC-NCA90 and SC-Sn-NCA90 before (a) and after 500th (b) cycle at 1 C between 2.8–4.5 V.

**Figure S26.** The detail models of Sn doping in the different position employed in DFT calculations.

**Figure S27**. The Li^+^ migration pathways for undoped NCA and Sn-doped NCA.

**Figure S28.** SEM images of NCA82 precursor (a and b) and Sn-NCA82 precursor (c and d). (e) EDS mapping of Sn-NCA82 precursor. SEM images of PC-NCA82 (f and g) and SC-Sn-NCA82 (h and i).

**Figure S29.** SEM images of NCA83 precursor (a and b) and Sn-NCA83 precursor (c and d). (e) EDS mapping of Sn-NCA83 precursor. SEM images of PC-NCA83 (f and g) and SC-Sn-NCA83 (h and i).

**Figure S30.** SEM images of NCA90-2 precursor (a and b) and Sn-NCA90-2 precursor (c and d). (e) EDS mapping of Sn-NCA90-2 precursor. SEM images of PC-NCA90-2 (f and g) and SC-Sn-NCA90-2 (h and i).

**Figure S31.** SEM images of NCA91 precursor (a-b) and Sn-NCA91 precursor (c-d). (e) EDS mapping of Sn-NCA91 precursor. SEM images of PC-NCA91 (f-g) and SC-Sn-NCA91 (h-i).

**Figure S32.** Electrochemical performance comparison of PC-NCA82 and SC-Sn-NCA82 cathodes. Initial charge-discharge at 0.05 C (a), rate performance (b), and cycling stability at 1 C (c) between 2.8–4.5 V. Initial charge-discharge at 0.05 C (d), rate performance (e), and cycling stability at 1 C (f) between 2.8–4.7 V.

**Table S1.** Lattice parameters of NCA90 precursor sintered at various temperatures for 24 h by Rietveld refinement.

| Sample | a/Å | c/Å | V/Å | Ni in Li layer (%) | *R*_wp_ | *R*_p_ | *χ*^2^ |
| --- | --- | --- | --- | --- | --- | --- | --- |
| 750 ^o^C | 2.870864 | 14.186998 | 101.262 | 2.45 | 2.16% | 1.62% | 1.866 |
| 800 ^o^C | 2.873661 | 14.188642 | 101.471 | 4.48 | 2.20% | 1.63% | 1.956 |
| 850 ^o^C | 2.876439 | 14.192978 | 101.698 | 5.69 | 2.29% | 1.69% | 2.105 |
| 900 ^o^C | 2.879909 | 14.203214 | 102.018 | 6.97 | 1.61% | 0.97% | 2.246 |

**Table S2.** Lattice parameters of Sn-NCA90 precursor sintered at various temperatures for 24 h.

| Temperature | a/Å | c/Å | V/Å | Ni in Li layer (%) | *R*_wp_ | *R*_p_ | *χ*^2^ |
| --- | --- | --- | --- | --- | --- | --- | --- |
| 700 ^o^C | 2.874523 | 14.200354 | 101.616 | 5.42 | 2.38% | 1.75% | 2.232 |
| 750 ^o^C | 2.873562 | 14.193310 | 101.498 | 2.44 | 2.20% | 1.68% | 1.885 |
| 800 ^o^C | 2.874523 | 14.190076 | 101.542 | 3.79 | 2.35% | 1.71% | 2.255 |

### **Table S3.** ICP-OES analysis of NCA90 precursor, PC-NCA90 and SC-Sn-NCA90.

| Sample | Li | Ni | Co | Al | Sn |
| --- | --- | --- | --- | --- | --- |
| NCA90 precursor | / | 0.8949 | 0.0862 | 0.0189 | / |
| PC-NCA90 | 1.0111 | 0.8945 | 0.0868 | 0.0187 | / |
| SC-Sn-NCA90 | 1.0174 | 0.8946 | 0.0868 | 0.0187 | 0.0023 |

### **Table S4.** ICP-OES analysis of various NCA precursor.

| Sample | Ni | Co | Al |
| --- | --- | --- | --- |
| NCA82 precursor | 0.8210 | 0.1578 | 0.0212 |
| NCA83 precursor | 0.8318 | 0.1123 | 0.0559 |
| NCA90-2precursor | 0.9008 | 0.0634 | 0.0357 |
| NCA91 precursor | 0.9128 | 0.0405 | 0.0467 |

### **Supplementary References**

1. J. Chen, G. Zou, W. Deng, et al., “Pseudo‐Bonding and Electric‐Field Harmony for Li‐Rich Mn‐Based Oxide Cathode,” *Advanced Functional Materials* 30 (2020): 2004302, https://doi.org/10.1002/adfm.202004302.

2. H. Tüysüz, C. Weidenthaler, T. Grewe, E.L. Salabaş, M.J.B. Romero, and F. Schüth, “A Crystal Structure Analysis and Magnetic Investigation on Highly Ordered Mesoporous Cr_2_O_3_,” *Inorganic Chemistry* 51 (2012): 11745-11752, https://doi.org/10.1021/ic301671a.

3. L.K. Alexander, N. Büttgen, R. Nath, A.V. Mahajan, and A. Loidl, “^7^Li NMR studies on the triangular lattice system LiCrO_2_,” *Physical Review B* 76 (2007): 064429, https://doi.org/10.1103/PhysRevB.76.064429.

4. T. Liu, S.-X. Zhao, K. Wang, and C.-W. Nan, “CuO-coated Li[Ni_0.5_Co_0.2_Mn_0.3_]O_2_ cathode material with improved cycling performance at high rates,” *Electrochimica Acta* 85 (2012): 605-611, https://doi.org/10.1016/j.electacta.2012.08.101.

5. A.S. Prakash, D. Larcher, M. Morcrette, M.S. Hegde, J.-B. Leriche, and C. Masquelier, “Synthesis, Phase Stability, and Electrochemically Driven Transformations in the LiCuO_2_−Li_2_CuO_2_ System,” *Chemistry of Materials* 17 (2005): 4406-4415, https://doi.org/10.1021/cm0508266.

6. A.T. Salih, S.H. Lafta, A.A. Najim, N.B. Mahmood, and G.K. Salman, “Structural, morphological and electrical properties of α-Fe_2_O_3_ nanoparticles prepared by chemical route,” *Ceramics International* 51 (2025): 25250-25257, https://doi.org/10.1016/j.ceramint.2025.03.207.

7. S.-P. Guo, Z. Ma, J.-C. Li, and H.-G. Xue, “First investigation of the electrochemical performance of γ-LiFeO_2_ micro-cubes as promising anode material for lithium-ion batteries,” *Journal of Materials Science* 52 (2016): 1469-147, https://doi.org/10.1007/s10853-016-0441-3.

8. L.-H. Yang, H.-X. Dong, Z. Sun, L.-X. Sun, X.-C. Shen, and Z.-H. Chen, “Temperature-Induced Phase Transition of In_2_O_3_ from a Rhombohedral Structure to a Body-Centered Cubic Structure,” *Chinese Physics Letters* 28 (2011): 087803, https://doi.org/10.1088/0256-307x/28/8/087803.

9. K. Xu, D. Xu, X. Zhang, Z. Luo, Y. Wang, and S. Zhang, “Visible-light activity of N-LiInO_2_: Band structure modifications through interstitial nitrogen doping,” *Applied Surface Science* 391 (2017): 645-653, https://doi.org/10.1016/j.apsusc.2016.07.037.

10. G. Xie, Z. Xu, D. Qian, et al., “Synthesis of Single‐Crystalline Ni‐Rich Cathodes for Lithium‐Ion Batteries With Superior High‐Voltage Stability by Mo/Nb Dual Doping,” *Energy & Environmental Materials* 0 (2026): e70319, https://doi.org/10.1002/eem2.70319.

11. J. Zhu, Y. Zhu, W. Shen, et al., “Growth and characterization of yttrium oxide films by reactive magnetron sputtering,” *Thin Solid Films* 519 (2011): 4894-4898, https://doi.org/10.1016/j.tsf.2011.01.049.

12. W. Yu, Y. Wang, A. Wu, et al., “Suppress oxygen evolution of lithium-rich manganese-based cathode materials via an integrated strategy,” *Green Energy & Environment* 9 (2024): 138-151, https://doi.org/10.1016/j.gee.2022.06.001.

13. N. Machida, J. Kashiwagi, M. Naito, and T. Shigematsu, “Electrochemical properties of all-solid-state batteries with ZrO_2_-coated LiNi_1/3_Mn_1/3_Co_1/3_O_2_ as cathode materials,” *Solid State Ionics* 225 (2012): 354-358, https://doi.org/10.1016/j.ssi.2011.11.026.

14. J. Zhang, H. Zhang, R. Gao, Z. Li, Z. Hu, and X. Liu, “New insights into the modification mechanism of Li-rich Li_1.2_Mn_0.6_Ni_0.2_O_2_ coated by Li_2_ZrO_3_,” *Physical Chemistry Chemical Physics* 18 (2016): 13322-13331, https://doi.org/10.1039/c6cp01366j.

15. C. Zeng, R. Zheng, F. Fan, et al., “Phase compatible surface engineering to boost the cycling stability of single-crystalline Ni-rich cathode for high energy density lithium-ion batteries,” *Energy Storage Materials* 72 (2024): 103788, https://doi.org/10.1016/j.ensm.2024.103788.

16. Y. Chu, G. Liang, Y. Mu, et al., “Enhanced Structural Stability of Single-Crystalline Ni-Rich Cathode Enables Improved Cyclability in Pouch Cells,” *ACS Nano* 19 (2025): 13842-13853, https://doi.org/10.1021/acsnano.4c16911.

17. Y. Zhou, H. Zhang, Y. Wang, et al., “Relieving Stress Concentration through Anion–Cation Codoping toward Highly Stable Nickel-Rich Cathode,” *ACS Nano* 17 (2023): 20621-20633, https://doi.org/10.1021/acsnano.3c07655.

18. J. Li, H. Yang, Q. Deng, et al., “Stabilizing Ni‐rich Single‐crystalline LiNi_0.83_Co_0.07_Mn_0.10_O_2_ Cathodes using Ce/Gd Co‐doped High‐entropy Composite Surfaces,” *Angewandte Chemie International Edition* 63 (2024): e202318042, https://doi.org/10.1002/anie.202318042.

19. Y. Huang, R. Tang, F. Xiao, L. Zeng, Y. Wang, and H. Huang, “Elevated rate and cycling performance of nickel-rich single-crystal at high voltage enabled by Al/Ce co-doping,” *Journal of Power Sources* 597 (2024): 234133, https://doi.org/10.1016/j.jpowsour.2024.234133.

20. H. Zhang, L. Qin, M. Sedlacik, et al., “Enhanced Li-ion intercalation kinetics and lattice oxygen stability in single-crystalline Ni-rich Co-poor layered cathodes,” *Journal of Materials Chemistry A* 12 (2024): 3682-3688, https://doi.org/10.1039/d3ta07156a.

21. X. Ou, T. Liu, W. Zhong, et al., “Enabling high energy lithium metal batteries via single-crystal Ni-rich cathode material co-doping strategy,” *Nature Communications* 13 (2022): 2319, https://doi.org/10.1038/s41467-022-30020-4.

22. Z. Xu, X. Chen, W. Fan, et al., “High-Entropy Rock-Salt Surface Layer Stabilizes the Ultrahigh-Ni Single-Crystal Cathode,” *ACS Nano* 18 (2024): 33706-33717, https://doi.org/10.1021/acsnano.4c13911.

23. Y. Wang, C. Chen, S. Weng, C. Li, Y. Xia, and X. Lu, “Stabilizing ultra-high Ni layered cathodes for high-voltage operation through Al doping with synergistic Sn modification,” *Chemical Engineering Journal* 514 (2025): 163172, https://doi.org/10.1016/j.cej.2025.163172.

24. X.a. Zhou, F. Zhang, X. Fu, et al., “Utilizing fast ion conductor for single-crystal Ni-rich cathodes to achieve dual-functional modification of conductor network constructing and near-surface doping,” *Energy Storage Materials* 52 (2022): 19-28, https://doi.org/10.1016/j.ensm.2022.07.029.

25. B. Cao, H.-T. Fang, D. Li, and Y. Chen, “Improved high-voltage cycling stability of single-crystalline LiNi_0.8_Co_0.1_Mn_0.1_O_2_ cathode by tantalum doping,” *Journal of Materials Chemistry A* 12 (2024): 28363-28373, https://doi.org/10.1039/d4ta04307c.

26. H. Bai, K. Yuan, C. Zhang, et al., “Advantageous surface engineering to boost single-crystal quaternary cathodes for high-energy-density lithium-ion batteries,” *Energy Storage Materials* 61 (2023): 102879, https://doi.org/10.1016/j.ensm.2023.102879.

27. P. Liu, H. Wei, T. Huang, et al., “Structural Regulation Enables High Interfacial Functionality for Ni-Rich Single-Crystalline Cathodes,” *ACS Applied Materials & Interfaces* 16 (2024): 53740-53749, https://doi.org/10.1021/acsami.4c10398.

28. J. Peng, J. Yang, S. Hao, et al., “Enhanced mechanical property promote high stability of single-crystal Ni-rich cathode at 4.5 V,” *Energy Storage Materials* 77 (2025): 104199, https://doi.org/10.1016/j.ensm.2025.104199.

29. Z. Tan, X. Chen, J. Lin, et al., “Restraining Planar Gliding in Single‐Crystalline LiNi_0.9_Co_0.05_Mn_0.05_O_2_ Cathodes by Combining Bulk and Surface Modification Strategies,” *Angewandte Chemie International Edition* 64 (2024): e202419903, https://doi.org/10.1002/anie.202419903.

30. Z. Tan, Y. Li, C. Lei, et al., “In Situ Constructing Ultrastable Mechanical Integrity of Single‐Crystalline LiNi_0.9_Co_0.05_Mn_0.05_O_2_ Cathode by Interior and Exterior Decoration Strategy,” *Small* 20 (2023): 202305618, https://doi.org/10.1002/smll.202305618.

31. Z. Tan, Y. Li, X. Xi, et al., “Construction of Planar Gliding Restriction Buffer and Kinetic Self-Accelerator Stabilizing Single-Crystalline LiNi_0.9_Co_0.05_Mn_0.05_O_2_ Cathode,” *ACS Applied Materials & Interfaces* 15 (2023): 8555-8566, https://doi.org/10.1021/acsami.2c22815.

32. W. Tang, Z. Shu, A. Li, X. Huang, and W. Li, “Enhancing structural integrity and long-term cycling stability of high-voltage single-crystalline Ni-rich cathodes via surface/subsurface dual-functional modification engineering,” *Energy Storage Materials* 77 (2025): 104185, https://doi.org/10.1016/j.ensm.2025.104185.

33 R. Wang, Y. Zhang, Z. Li, et al., “Improving structure stability of single-crystalline Ni-rich cathode at high voltage by element gradient doping and interfacial modification,” *Journal of Energy Chemistry* 101 (2025): 630-640, https://doi.org/10.1016/j.jechem.2024.10.015.

34. H. Zhang, H. Yu, E. Zhang, Q. Cheng, Q. Cheng, and H. Jiang, “One-step surface-to-bulk modification of single-crystalline Ni-rich Co-poor cathodes for high-rate and long-life Li-ion batteries at high-voltage operations,” *Chemical Engineering Journal* 498 (2024): 155617, https://doi.org/10.1016/j.cej.2024.155617.
